# Chloride Channels as Determinants of Anti-Cancer Drug Response: Why the Therapeutic Direction Reverses Between Channels and Between Tissues

**DOI:** 10.3390/cancers18203369

**Published:** 2026-10-18

**Authors:** Yohan Seo

**Affiliations:** Department of Physiology, Dongguk University College of Medicine, 123 Dongdae-ro, Gyeongju-si 38066, Gyeongsangbuk-do, Republic of Korea; ddukdae12@dongguk.ac.kr; Tel.: +82-54-770-2416

**Keywords:** chloride channel, drug resistance, LRRC8, VRAC, CLIC1, ClC-3, ANO1/TMEM16A, CFTR, cisplatin, drug repurposing

## Abstract

**Background/Objectives:** Resistance to cytotoxic chemotherapy and radiotherapy is usually attributed to DNA repair, drug efflux and apoptotic threshold. Chloride channels intersect these processes but are rarely considered as a group. This review asks whether chloride flux forms a coherent, targetable axis of treatment response and, if not, how the therapeutic direction differs by channel and tissue. **Methods:** PubMed was searched to 23 August 2026; all free-text searches were re-executed on 22 September 2026 and supplemented by controlled-vocabulary searches. Cited records were checked against NCBI E-utilities and screened for retractions. Claims were graded by whether pharmacological phenotypes were reproduced by genetic perturbation and by the available target-engagement and replication evidence. **Results:** The therapeutic direction is not uniform. In experimental models, LRRC8A- and LRRC8D-containing channels mediate substantial platinum uptake, and dose-stratified associations between their expression and outcome have been reported in head and neck cancer, supporting prospective evaluation as predictive biomarkers; contrary reports in ovarian, colon and breast models indicate that this direction is established for platinum uptake rather than for cytotoxic resistance in general. CFTR is an intestinal tumour suppressor and the only channel reviewed with an approved potentiator, yet its direction reverses in glioma, with a prostate report of CFTR-dependent cisplatin resistance pointing in the same direction. Conversely, ClC-3, ANO1 and CLIC1 are associated with resistance when gained, although CLIC1 is better supported as a determinant of drug response than of tumorigenesis. Sixteen modulators are compared by potency, assay format, selectivity/counter-screening and clinical status; widely used blockers are often poorly selective. **Conclusions:** Chloride transport is not a single therapeutic target class. Candidate chloride-channel blockers should be counter-screened against LRRC8/VRAC before combination with platinum therapy, and CFTR potentiation should not be generalised across tumour types. Conflicting evidence is reported alongside the directions it qualifies rather than set aside.

## Simple Summary

Cancer cells that stop responding to chemotherapy are often explained by DNA repair or pumps that expel the drug. A less familiar contributor is chloride-ion movement across membranes. Chloride-transporting proteins set cell volume, influence organellar acidity, and in some cases form part of the route by which a drug enters the cell. This review examines evidence that five such proteins alter responses to platinum drugs, temozolomide or radiotherapy. They do not point in the same therapeutic direction: blocking some may improve treatment response, whereas blocking others could worsen it, and one protein points in opposite directions in different tissues. We also examine why commonly used small-molecule blockers are unreliable and grade claims according to whether the phenotype persists when the proposed channel target is removed genetically.

## 1. Introduction

Ion channels have moved from the periphery of cancer biology toward its centre over the past two decades, but the attention has been unevenly distributed. Potassium and calcium channels dominate the literature, while chloride channels—despite carrying the largest anionic conductance in most cells and having an established role in the volume changes that accompany both proliferation and apoptosis—are usually treated as supporting players.

This is a curious omission for a field concerned with drug resistance, because chloride channels sit at three separate points in the causal chain that determines whether a cytotoxic agent works. They can form part of the route by which a drug enters the cell. They govern the regulatory and apoptotic volume changes that decide whether a damaged cell dies or persists. And, through their contribution to organellar acidification and to transcriptional programmes controlling efflux machinery, they influence how much drug is sequestered or exported once inside.

Whether these three points add up to a single, coherent chloride axis is the question this review sets out to answer, and the answer is a qualified no. The evidence does not support a unified prescription. It supports a directional split: for some channels, the therapeutic logic is to block, while for others it is the opposite. Conflating them carries a specific and foreseeable clinical risk that is set out in Section 6.

Two prior reviews in this journal have examined the anoctamin family, in relation both to calcium signalling as an obstacle to EGFR-targeted therapy in glioblastoma [1] and to cell survival and death [2], and a recent atlas has surveyed transmembrane protein families in lung cancer [3]. Each treats a family from within. Chloride transport and treatment response have also been reviewed directly: as a survey of volume-regulated and calcium-activated anion channels in cell volume homeostasis, cancer and drug resistance [4]; as an account of the volume-sensitive outwardly rectifying and maxi-anion channels in apoptosis and cisplatin resistance [5]; as a channel-by-channel description of chloride conductances in primary brain tumours [6]; and as a treatment of chloride channels in multidrug resistance [7]. Those accounts establish that the association exists and they remain the better sources for biophysics. None of them asks in which direction intervention should act, none sets the proteins against one another on that axis, and none grades a claim by whether the phenotype survives removal of the protein. This review places anoctamins as one entry among several chloride-conducting families, compares directions of effect across them, and applies that grading standard throughout.

This review has three objectives. The first is to determine, for each chloride-transporting protein with sufficient cancer evidence, whether the data support blockade or preservation of function as the therapeutic direction. The second is to establish whether that direction is a property of the protein or of the tissue in which it is expressed. The third is to define an evidence standard by which claims in this literature can be graded, given that the compounds used to generate them are frequently non-selective. Four factors are assessed against each protein: the mechanism by which chloride flux alters drug effect; whether the pharmacological phenotype is reproduced by genetic loss of the protein; the tissue context in which the effect was observed; and the selectivity evidence available for any modulator involved. Section 3, Section 4 and Section 5 develop the evidence, Section 6 resolves the directionality problem, Section 7 sets out the grading standard, and Section 8 applies it to reported modulators. We have previously proposed a staged decision architecture for ion-channel drug discovery, concerned with how candidates should progress through screening platforms [8]; this review addresses a different question for a set of anion-transporting proteins, namely, in which direction a candidate should act.

## 2. Search Strategy and Evidence Grading

This is a narrative review. The search is reported below, and in full in Supplementary Table S1, so that its scope and boundaries are explicit.

The literature was identified through PubMed using combinations of channel names (LRRC8*, VRAC, CLIC1, *CLCN3*/ClC-3, *ANO1*/TMEM16A, *ANO6*/TMEM16F, *CFTR*, bestrophin) with terms for neoplasia and treatment response (cancer, drug resistance, chemoresistance, cisplatin, radiotherapy), supplemented by backward citation chaining from the reviews retrieved. No lower limit on publication date was applied, except where stated for individual searches in Supplementary Table S1; the cited literature spans 2004 to 2026. The final free-text search was executed on 23 August 2026. During revision, every search string was rerun on 22 September 2026 and the strategy was supplemented with four controlled-vocabulary (MeSH) searches constructed independently of the free-text strings to test the strategy for concept coverage rather than to extend its date range. The additional strings, the record counts from both rounds and the records added as a result are documented in Supplementary Table S1. Verbatim search strings and the operational inclusion and exclusion criteria are provided in the Supplementary Materials. All cited records were verified programmatically against NCBI E-utilities for bibliographic accuracy and screened for retractions and expressions of concern; none were retracted.

Studies were included if they reported a chloride channel or its modulator together with a neoplastic phenotype or treatment-response endpoint and provided at least one of the following: genetic perturbation, direct current or flux measurement, structural data, animal efficacy, or a human clinical correlate. Records reporting only non-neoplastic phenotypes were excluded, as were expression correlations for which a stronger source existed for the same claim. On re-execution, the fifteen free-text searches returned 3448 search hits in aggregate, of which 3307 came from a single broad surveillance sweep screened by title in relevance order to the point of conceptual saturation rather than exhaustively; the four controlled-vocabulary searches returned 151 additional search hits in aggregate before assessment of overlap. Nine records that met the criteria above but had not been retrieved by the original free-text strategy were identified in this way and incorporated. Before resubmission, the strategy was audited a third time, specifically for concept coverage rather than for currency: searches were built from protein names absent from the original string set, from MeSH pairings of chloride channels with neoplastic drug resistance, and from the four protein-specific concepts carrying the directional argument. That audit returned a further fourteen records meeting the criteria above, several of which report effects in the opposite direction to those claimed in the submitted version; all fourteen are cited, and the contrary evidence is set out where it applies rather than confined to the limitations. The revised manuscript cites 127 works in total, including contextual sources used to establish channel identity, structure or general physiology; the operational eligibility criteria above apply to the claim-bearing evidence synthesis rather than to those contextual citations. Records that initially appeared eligible but were excluded, and the ground for each exclusion, are listed in Supplementary Table S1. Each claim was then graded by the standard set out in Section 7.

## 3. The Chloride-Channel Landscape in Cancer

The proteins compared in this review are routinely grouped as chloride channels, but they do not constitute a single family. LRRC8 heteromers, the TMEM16/anoctamin proteins, the CLC transporters, the metamorphic CLIC proteins and *CFTR* differ in fold, in gating and in whether they conduct anions at all. They are grouped here by the phenotype they influence, not by ancestry, and the grouping is a convenience of the clinical question rather than a statement about structure.

The calcium-activated chloride channel *ANO1*/TMEM16A was identified in 2008 as the molecular basis of the long-sought calcium-activated chloride current [9], and the family it defines was initially proposed as a new class of chloride channels [10]. That framing did not survive contact with the individual members. Reconstitution in a scramblase-null background showed that most support calcium-dependent phospholipid scrambling rather than chloride conduction [11], and the first crystal structure of a fungal homologue revealed the open, membrane-facing groove through which lipid headgroups pass [12,13]. Whether all ten members are chloride channels was questioned early [14] and the physiological picture was consolidated soon after [15], with successive reviews acknowledging that structure and function in this family remained genuinely confusing [16]. High-resolution cryo-EM of TMEM16A subsequently resolved how calcium binding opens the sealed pore [17], and later work treated lipid scrambling and ion conduction as two outcomes of one architecture [18,19]. Members acting at intracellular membranes form a further subset [20,21].

*ANO6*/TMEM16F sits between the two behaviours, forming a calcium-activated cation channel required for lipid scrambling [22]. Its canonical role is in haemostasis, where scrambling supports phosphatidylserine exposure and microparticle release in activated platelets [23] and is required for normal thrombosis in mice [24]. That activity also explains why *ANO6* enters the cancer literature: phosphatidylserine exposure and microparticle release are the cellular events through which tumour cells influence coagulation, immune recognition and membrane repair; therefore, in oncology, *ANO6* is discussed as a scramblase rather than as a chloride conductance, and the evidence is assessed on that basis in Section 5.6.

The volume-regulated anion channel is a distinct molecular entity. Its molecular identity was established in 2014 by two independent genome-wide RNA interference screens: one identified *LRRC8A* as a VRAC component, showed that it forms heteromers with other LRRC8 proteins, and found that cells lacking all five LRRC8 genes required *LRRC8A* together with another isoform to reconstitute currents, with the isoform combination setting inactivation kinetics [25]; the other independently identified the same protein as SWELL1, localised it to the plasma membrane, and showed that point mutations alter VRAC anion selectivity, which is the observation that establishes it as a pore-forming rather than a regulatory subunit [26]. Its physiological remit extends well beyond volume regulation [27], and CLIC1 is not a conventional membrane channel but a protein that transitions between soluble and membrane-inserted states [28]. ClC-3, encoded by *CLCN3*, belongs to the CLC superfamily but is not a conventional plasma-membrane chloride channel: it functions predominantly as an intracellular electrogenic 2Cl^−^/H^+^ exchanger, with the bulk of the protein residing on endosomal and synaptic vesicle membranes rather than at the cell surface [29]. Whether its reported cancer phenotypes require exchanger activity, a minor plasma membrane conductance, or functions unrelated to transport remains unresolved, and this ambiguity should be acknowledged in all claims made about it below. Chloride channels as a group have been surveyed within gastrointestinal cancer [30], and the regulation of *ANO1*, in particular, has been reviewed repeatedly [31,32].

These proteins have entered the cancer literature through different routes, and the difference affect how the evidence should be interpreted. *ANO1* entered as a diagnostic antigen and only later as a target, so the literature on its role in cancer is dominated by expression and prognosis studies. LRRC8/VRAC entered through drug uptake, so the associated literature is dominated by membrane pharmacokinetics and has the most direct link to a treatment decision. *CFTR* entered through human genetics, which is why it is the only protein here with causal evidence from an inherited disease. CLIC1 entered the literature through the stem-like compartment of glioblastoma and through a repurposing hypothesis concerning metformin. ClC-3 entered through multidrug resistance and organellar acidification. *ANO6*, bestrophin-1 and the *ANO7*/*ANO9* pair entered through expression profiling alone. The order in which each protein acquired expression, functional and genetic evidence therefore differs, and Section 5.1, Section 5.2, Section 5.3, Section 5.4 and Section 5.5 examine each one in a consistent order so that those differences are visible rather than averaged away.

## 4. Five Mechanisms Linking Chloride Flux to Drug Response

Five mechanisms recur in this literature and are summarised in Figure 1. Each is set out below with the evidence that supports it, and the grade is assigned per mechanism rather than per protein, because the same protein is supported at different strengths for different mechanisms.

### 4.1. The Channel as a Drug Entry Route

This is the most direct and best-evidenced mechanism, and it belongs primarily to VRAC. Loss of *LRRC8A* or *LRRC8D* increases resistance to clinically relevant concentrations of cisplatin and carboplatin, and in the cell models used in that study, roughly half of isotonic cisplatin uptake depended on these two subunits but not on *LRRC8C* or *LRRC8E*, making the effect subunit-specific rather than a general property of the channel [33]. The proportion is specific to those models and assay conditions and should not be treated as a general constant. Cell swelling markedly enhances this uptake, and incorporating *LRRC8D* substantially increases permeability to both cisplatin and taurine, the observation that most directly supports the LRRC8 proteins forming the conducting pore rather than merely regulating it [33].

The causal direction has been investigated from the opposite perspective. In Xenopus oocytes, which lack endogenous VRAC activity, cisplatin exposure strongly activated currents through *LRRC8A*/8D and *LRRC8A*/8E heteromers, consistent with the drug both permeating and activating the channel. The authors of that study proposed LRRC8 activators as a means of counteracting resistance [34], inverting the therapeutic logic that applies to most other channels considered here. A second, more provisional candidate for this mechanism is *CFTR* (Section 5.5).

### 4.2. Apoptotic Volume Decrease

VRAC contributes through a second, uptake-independent route: *LRRC8A* disruption suppressed drug-induced apoptosis even after accounting for uptake, possibly through impaired apoptotic volume decrease [33]. The channel therefore has a dual role, as an entry route and an execution step. The attribution to apoptotic volume decrease is the original authors’ interpretation rather than a demonstrated causal step, and is treated as such throughout.

### 4.3. Organellar Acidification and Drug Sequestration

Vesicular acidification is driven by the V-ATPase; ClC-3-mediated Cl^−^/H^+^ exchange can modify the vesicular electrical gradient and luminal chloride concentration rather than acidify the compartment itself. On that basis, it has been proposed that ClC-3 increases sequestration of weakly basic drugs [29]. Direct experimental support for the sequestration step exists and predates that proposal. In neuroendocrine tumour lines and in HEK293 cells, ClC-3 was localised to LAMP-1-positive late endosomal and lysosomal vesicles; its overexpression increased intravesicular acidity and approximately doubled the half-maximal inhibitory concentration of etoposide, and inhibition of the V-ATPase reduced that resistance. Neither P-glycoprotein nor multidrug-resistance-associated protein 1 was detectable in the parental line, which excludes the efflux-pump explanation for the effect [35]. Two limits keep this below the top of the scale. The design is gain-of-function, so the phenotype has not been shown to be reproduced by loss of ClC-3; and etoposide is not a weakly basic drug. Therefore, the result supports acidification-dependent sequestration in general without establishing the weak-base ion-trapping mechanism specifically proposed for this channel. Transport causality was not tested. The claim is graded accordingly.

### 4.4. Transcriptional Coupling to Efflux Machinery

ClC-3 was highly expressed in P-glycoprotein-dependent paclitaxel-resistant A549 and doxorubicin-resistant MCF-7 cell lines. Manipulation of ClC-3 converted sensitive cells into resistant cells and vice versa. ClC-3 upregulated ABCB1 (*MDR1*) messenger RNA and its product P-glycoprotein through NF-κB. Furthermore, double-transgenic MMTV-PyMT/*CLCN3* mice with spontaneous mammary tumours and ClC-3 overexpression were resistant to both paclitaxel and doxorubicin [36]. A parallel axis places ClC-3 downstream of a stemness factor: *SOX2* bound the ClC-3 promoter and enhanced its transcription, and knockdown of either reduced paclitaxel resistance [37]. Bidirectional manipulation, together with an in vivo genetic model, is strong evidence; the limitation is that both axes rest largely on the same two cell-line pairs without independent laboratory replication.

### 4.5. Maintenance of the Stem-like Compartment

CLIC1 was overexpressed in glioblastoma relative to normal brain, with better survival among low-expressing tumours. Within patient-derived neurospheres, it was enriched in the stem and progenitor compartment, and silencing reduced proliferative, clonogenic and tumorigenic capacity [38]. Critically for a channel claim, reducing CLIC1 chloride current with a specific antibody reproduced the silencing phenotype [38], which is functional rather than merely expression-based evidence. The grade is nonetheless kept below the maximum because the work originates predominantly from one collaborating network, and because CLIC1 exists in both soluble monomeric and membrane-inserted dimeric forms whose ratio varies with redox state [28]. Consequently, CLIC1 expression and CLIC1 channel activity are not interchangeable readouts. Extracellular vesicle-mediated transfer of the protein adds a further layer to how the compartment is sustained [39].

## 5. Channel-by-Channel Synthesis

Five proteins carry evidence sufficient to support a directional argument and are treated as such below: LRRC8/VRAC, CLIC1, ClC-3, *ANO1*/TMEM16A and *CFTR*. Three further entries—*ANO6*/TMEM16F, bestrophin-1, and the *ANO7*/*ANO9* pair—are included because omitting them would misrepresent the field, but they are presented as unresolved, insufficient, or non-directional, respectively, and no therapeutic direction is inferred from them. Section 5.1, Section 5.2, Section 5.3, Section 5.4 and Section 5.5 all follow the same order: how the protein behaves biologically, what associates it with cancer, what the resistance or treatment-response evidence shows, what pharmacology exists, and what therapeutic direction the evidence supports.

### 5.1. LRRC8/VRAC: The Resistance Direction Is Inverted

VRAC carries the only clinical correlative dataset in this review that bears directly on a treatment decision. In head and neck squamous cell carcinoma treated with platinum-based chemoradiotherapy, the prognostic value of *LRRC8A* and *LRRC8D* emerged only once patients were stratified by cumulative cisplatin dose. No formal test of statistical interaction was reported, so the finding is best described as a stratified association that differed between dose groups. When patients were divided at the established 200 mg/m^2^ cumulative threshold, those with high *LRRC8A* expression appeared likely to benefit from the higher dose, whereas loss of *LRRC8A* expression flattened the survival curves toward those of the lower, less effective dose group. The authors accordingly proposed that *LRRC8A* and *LRRC8D* should be incorporated into a prospective trial to predict the success of intensified platinum therapy [40]. The same study reported that deletion of the mouse gene *Lrrc8d* did not affect mouse viability or fertility and that such mice tolerated twice the cisplatin maximum tolerable dose, enabling a model of intensified therapy [40].

Mechanistic granularity continues to accumulate. *NAA60*, a Golgi-localised N-terminal acetyltransferase, was identified as a factor whose loss decreases platinum uptake and confers resistance in otherwise hypersensitive BRCA1;p53-deficient cells and tumours, with the data suggesting acetylation of the *LRRC8A* and *LRRC8D* N-termini as the mechanism [41].

Two further lines of evidence supports the same interpretation. In head and neck squamous cell carcinoma, a bioinformatic screen of candidate resistance factors across 565 patients identified the volume-regulated anion channel as the component associated with recurrent disease after radiochemotherapy, ahead of the copper transporter CTR1, the organic cation transporter OCT1 and multidrug-resistance-associated protein 1. CRISPR/Cas9 knockout produced cisplatin-resistant cells, and re-expression restored sensitivity [42]. This is the same tumour type and direction as with the dose-stratified dataset above, reached by a different group with a genetic rather than a correlative design, and it represents the closest approximation to independent replication applied in the clinical side of this argument. Separately, the puromycin-sensitive aminopeptidase *NPEPPS* was found to interact with the channel and to restrain cisplatin import, with the *NPEPPS*-to-VRAC expression ratio behaving as a predictor of cisplatin response across several tumour cohorts [43]. This reframes the biomarker proposal as a ratio between an importer and a negative regulator of it rather than as a single-protein measurement, which is a more tractable object for prospective testing.

The direction is nonetheless not uniform across drugs and models, and the contrary reports are set out here rather than left for a reader to find. In ovarian carcinoma, acquired cisplatin resistance was accompanied by reduced *LRRC8A* and CTR1 expression, together with increased ATP7A and ATP7B expression; platinum accumulation tracked *LRRC8A* protein and channel activity; and the authors described a dual role for *LRRC8A*-containing transporters in resistance [44]. In colon cancer, *LRRC8A* was raised rather than lost in an oxaliplatin-resistant derivative and was reported to promote the early development of that resistance [45]. In breast cancer spheroids, *LRRC8A* expression rose relative to the monolayer culture and its pharmacological inhibition reduced resistance to doxorubicin, gemcitabine and 5-fluorouracil, with downregulation of multidrug-resistance-associated protein 3 and of cytochrome P450 3A4 proposed as the mechanism [46]. These findings are not trivially reconcilable with the platinum account, and three features distinguish them. The drugs are not all platinum compounds, whereas the uptake argument is specific to platinum permeation through the pore. Two of the three measure expression in a resistant derivative rather than manipulate the channel in a sensitive one, so the direction of causation is not fixed. And the spheroid result attributes the effect to transporter and enzyme expression rather than to anion conduction, with no conduction-deficient control. The defensible reading is that the preserve-or-activate direction is established for platinum uptake in the models where uptake was measured directly, and that *LRRC8A* carries additional, drug- and context-dependent roles that are not uptake-mediated. The recommendation in Section 6 is therefore stated for platinum specifically and not for cytotoxic therapy in general.

The intervention implied is therefore not a blocker. Rather, it involves the use of *LRRC8A* and *LRRC8D* expression as candidate predictive biomarkers for platinum benefit, which would require prospective validation, and LRRC8 activation as a sensitisation strategy [34]. Pharmacological activation remains hypothetical, and any sensitisation strategy would require tumour-selective delivery or a demonstrable tumour-to-normal-tissue window, because enhanced LRRC8-dependent platinum uptake would be expected to increase normal-tissue toxicity as readily as tumour killing. No selective LRRC8 activator suitable for in vivo use was identified in this search, and this is a concrete medicinal-chemistry gap rather than a general observation about the field. The biomarker proposal is the better-supported half of this recommendation; the activation strategy rests on a single heterologous-expression study and is retained as a hypothesis.

### 5.2. CLIC1: The Clearest Repurposing Narrative, and Its Correction

The identification of CLIC1 as the antitumour target of metformin rests on an unusually specific line of evidence. In U87 glioblastoma cells, the CLIC1 inhibitor IAA94 and metformin were non-additive in perforated patch-clamp recordings and their current–voltage relationships superimposed, indicating convergence on a common target; metformin inhibited CLIC1 with a half-maximal effective concentration of 2.1 mM compared to 32 µM for IAA94 [47]. That potency gap is the central translational problem of the CLIC1 programme, and subsequent work can be read as an attempt to close it. Inhibition proved to be a biguanide class effect, with phenformin, cycloguanil, moroxydine and proguanil acting through the same mechanism and differing in the balance of efficacy, potency and off-target toxicity [48]. Target engagement was then established at residue resolution: a point mutation at arginine 29 abolishes the antitumour effect of metformin, supported by single-channel patch-clamp and nuclear magnetic resonance data, with a downstream route through *CIP2A* inhibition, protein phosphatase 2A B56δ activation, GSK3β dephosphorylation and MCL-1 breakdown under hypoglycaemic conditions [49].

A ligand-based search then abandoned the biguanide scaffold and identified the diuretic triamterene as a CLIC1 blocker with a half-maximal inhibitory concentration reported as roughly 200-fold lower than that of metformin [50]. Triamterene reduced stem-cell proliferation in two- and three-dimensional culture, decreased invasiveness, showed antitumour activity in zebrafish xenografts, and increased temozolomide potency and the cytotoxicity of combined radiation plus temozolomide, while producing only a modest viability reduction in immortalised astrocytes, oligodendrocytes and brain endothelial cells [50]. Because the absolute potency was not recoverable from the accessible text, and because the metformin value above was obtained in a different cell system, the two are reported here as a ratio, rather than combined into an absolute figure.

A qualification must be recorded alongside this narrative. CLIC1 activity is not required for glioblastoma development, even though its inhibition dictates the responsiveness of glioma stem cells to novel biguanide derivatives [51]. This is not a contradiction, but it changes how the target should be framed: CLIC1 appears to be a determinant of drug response rather than a driver of tumorigenesis, and a blocker would accordingly be expected to function as a sensitiser used in combination—which is how triamterene behaved with temozolomide and radiation—rather than as a single agent capable of arresting an established tumour.

Two studies published since then extend CLIC1 beyond the network and the tumour type on which the account above rests. In cisplatin-resistant AGS and MKN28 gastric cancer lines, CLIC1 expression was increased relative to the parental lines and autophagy was activated; CLIC1 overexpression reduced cisplatin sensitivity, whereas CLIC1 silencing or autophagy inhibition restored it [52]. This is a different tumour type, a different downstream mechanism, and an unconnected group, which supports the claim that CLIC1 is a determinant of drug response. The evidence is expression- and silencing-based, without current measurement, so it does not by itself raise the grade.

The second study addresses the engagement question directly. Mapping the oncostatin M receptor interactome in glioblastoma identified CLIC1 as a regulator of OSMR-STAT3 signalling and of the OSMR/EGFRvIII complex: genetic deletion of CLIC1 disrupted the OSMR/EGFRvIII interaction, impaired STAT3 activation, reduced EGFRvIII content in extracellular vesicles and slowed tumour progression, while whole-cell patch-clamp recording together with a monoclonal antibody directed at transmembrane CLIC1 established a tmCLIC1-mediated current required to sustain EGFRvIII/STAT3 signalling [53]. Genetic deletion, direct current measurement and a selective biological tool in one study constitute the combination that the standard of Section 7 asks for, and this places CLIC1 alongside ANO1 as a chloride-transporting protein that acts partly by organising EGFR-family signalling rather than only by conducting anions. It does not remove the reservation recorded above about the ratio of soluble to membrane-inserted CLIC1, which continues to make expression and channel activity non-interchangeable readouts. The study does, however, belong to the same collaborating network as the earlier glioblastoma work, and therefore strengthens the mechanistic case without supplying the independent replication that a higher grade would require.

The most recent addition to CLIC1 pharmacology comes from an unrelated direction. By using cysteine-reactive competitive activity-based protein profiling and surface plasmon resonance, CLIC1 was identified as a direct binding target of the flavonoid luteolin in hepatocellular carcinoma; luteolin inhibited proliferation and induced ferroptosis-associated changes, including reduced *GPX4* and *SLC7A11*, increased *ACSL4*, and elevated ferrous iron, reactive oxygen species and lipid peroxidation, which were partially reversed by ferroptosis inhibitors, with high CLIC1 expression associated with poor prognosis [54]. Luteolin has separately been shown to inhibit human *ANO1* in patch-clamp recordings and to suppress the calcium-activated chloride current in airway epithelial cells while leaving the *CFTR* current unaffected [55]. This is therefore the third documented case in which a chloride-channel-directed agent acts on more than one family member. Notably, luteolin has been counter-screened against *CFTR* and passed; it has not been tested against LRRC8/VRAC, which is precisely the gap identified in Section 6.

### 5.3. ClC-3: Mechanistically Rich, Pharmacologically Weak

ClC-3 carries the broadest set of proposed resistance mechanisms (Section 4.3 and Section 4.4), together with one chemosensitisation result: inhibition enhanced cisplatin sensitivity in SiHa cervical cancer cells and reversed resistance in the cisplatin-resistant SiHa/DDP line through Akt–mTOR-mediated autophagy, and a ClC-3-directed inhibitor sensitised cervical cancer xenografts to cisplatin in vivo [56]. The inhibitor used was chlorotoxin, which is described by those authors as ClC-3-specific. That description is not adopted here. A dedicated binding study using recombinant proteins and two independent bead-based assays set out to resolve the long-standing disagreement over what chlorotoxin actually binds, listing the ClC-3 chloride channel, matrix metalloproteinase 2, regulators of that enzyme, annexin A2 and neuropilin 1 among the targets previously proposed. Strong interaction was confirmed for matrix metalloproteinase 2 and neuropilin 1 with dissociation constants of roughly 0.5 to 0.7 µM; binding to annexin A2 was not confirmed; and chlorotoxin did not inhibit matrix metalloproteinase 2 activity [57]. Tool selectivity is therefore contradicted by confirmed MMP-2 and neuropilin 1 binding, and ClC-3 binding was not established by that study. A compound whose target assignment remains this unsettled cannot carry the weight of a channel-specific claim, and the associated result is graded accordingly.

The chemosensitisation result is not confined to the cervical models, and it has been reproduced elsewhere without the chlorotoxin confound. In cholangiocarcinoma, ClC-3 was elevated in chemoresistant tumour tissue and in the cisplatin-resistant QBC939/DDP line; ClC-3 knockdown increased cisplatin-induced cell death in both the parental and the resistant cells, and the autophagy agonist rapamycin abolished that gain without altering ClC-3 expression, placing autophagy downstream of the channel rather than beside it [58]. This is genetic rather than pharmacological manipulation, in a different tumour type and by an unconnected group. Therefore, the autophagy-linked chemosensitisation claim now carries independent replication even though the chlorotoxin-based in vivo arm does not. What neither study provides is a conduction-deficient construct, so whether chloride transport itself is the operative variable remains untested.

A computational study has since screened approximately 180,000 compounds from ZINC15 by molecular docking with three-dimensional convolutional neural network rescoring, ADMET filtering and 100-nanosecond all-atom molecular dynamics, converging on a single ligand and proposing a previously uncharacterised binding pocket within the ClC-3 transmembrane domain [59]. This updates the statement that no selective ClC-3 modulator has been proposed. It does not, however, satisfy the standard applied here, since no experimental data accompany the prediction; a docking pose is a hypothesis about a mechanism, not evidence of one.

### 5.4. ANO1/TMEM16A: The Broadest Cancer Footprint, and the Least Uniform

#### 5.4.1. Diagnostic Origin

*ANO1* entered oncology before it entered channel biology. Under the alias DOG1, its immunohistochemical positivity was established as a near-universal diagnostic marker for gastrointestinal stromal tumour, irrespective of *KIT* or *PDGFRA* mutation status [60]: this is a diagnostic role, not a therapeutic one, and one that still accounts for most clinical use of the protein.

#### 5.4.2. Prognostic Association Across Tumour Types

Overexpression has since been reported as an adverse prognostic indicator across a wide range of carcinomas: colorectal [61], lung, where *ANO1* protein was overexpressed in 77.3% of human lung adenocarcinoma specimens by immunohistochemistry and short hairpin RNA silencing suppressed proliferation, migration and invasion in two cell lines as well as xenograft growth [62]; oesophageal squamous cell carcinoma, where genome-wide copy-number analysis identified *ANO1* as an oncogene and prognostic biomarker [63]; ovarian carcinoma, where inhibition suppressed tumour growth through inactivation of PI3K/Akt signalling [64]; and glioma, where overexpression promoted tumour formation through NF-κB [65]. Oral squamous cell carcinoma has been approached pharmacologically on the same premise, with simvastatin reported to act on the tumour by targeting *ANO1* [66]. Cell-cycle entry has been linked to regulation of the channel by casein kinase 2 [67].

The direction is not uniform, and this should not be overlooked. In breast cancer stratified by receptor status, *ANO1* overexpression was associated with good prognosis in the progesterone-receptor-positive or HER2-negative subtype following tamoxifen treatment [68]. Any account presenting *ANO1* as a uniformly adverse marker is selecting from the literature rather than summarising it, and the subtype dependence anticipates the tissue dependence documented for *CFTR* in Section 5.5.

#### 5.4.3. Mechanistic Routes

Three mechanistic routes recur. The first is physical and functional coupling to EGFR: a proteomics screen showed that *ANO1* complexes with EGFR and that *ANO1* amplification correlates with sensitivity to EGFR-targeted agents in head and neck cancer [69], and in pancreatic cancer TMEM16A was shown to control EGF-induced calcium signalling in a manner implicated in prognosis [70]. The second is calcium-dependent signalling more broadly, as reviewed in the context of glioblastoma, where it has been argued to obstruct EGFR-targeted therapy [1]. The third is metabolic: *ANO1* reprograms cholesterol metabolism and the tumour microenvironment to promote metastasis [71], a mechanism distinct from its signalling roles and one that attracted immediate commentary for connecting liver X receptor signalling to metastatic behaviour [72]. Anoctamins have also been examined specifically for their contribution to cell survival and cell death [2], and the therapeutic case for *ANO1* across epithelium-derived disease has been examined at length [73].

#### 5.4.4. Resistance: Where the Evidence Is Cleanest

The resistance-specific evidence for *ANO1* is narrower than the prognostic literature but, in one respect, unusually clear. Across a panel of cell lines, *ANO1* messenger RNA expression was related to radiosensitivity measured as D0, the dose in Gy required to reduce surviving fraction to 37%, derived from clonogenic survival curves fitted to the linear-quadratic model. The radioresistant FaDu derivative showed a D0 approximately 45% higher than its radiosensitive counterpart: 2.89 against 1.98 Gy on average [74]. In an 11q13-amplified model of primary cisplatin resistance, silencing either *CCND1* or *ANO1* increased cisplatin sensitivity, yet only *ANO1* silencing increased radiosensitivity, with distinct pathway alterations—VEGF signalling for *CCND1* and the Rho GTPase cycle for *ANO1*—alongside shared changes in DNA damage response and cell-cycle regulation. In 415 HPV-negative cases from the TCGA head and neck cancer dataset, *CCND1* and *ANO1* messenger RNA levels were co-expressed, as expected of an amplicon (Pearson coefficient 0.64). However, roughly 9% of cases diverged, and it was high *ANO1* rather than high *CCND1* that was prismarily associated with poor overall survival. This pattern was reproduced in an independent institutional cohort treated with cisplatin-based radiochemotherapy, and copanlisib and afatinib were identified as candidates for combination [74].

The value of this study for the present review lies in what it excludes rather than what it shows. *ANO1* sits within the 11q13 amplicon alongside *CCND1*; therefore, co-amplification has confounded much of the older literature attributing phenotypes to *ANO1*. Dissecting the two by parallel knockdown, and finding that they diverge on radiosensitivity specifically, is the kind of control that the grading standard set out in Section 7 is designed to reward. The broader clinical significance of *ANO1* across neoplastic and non-neoplastic disease has been reviewed recently [75], as has its pharmacology within the digestive system, where the selectivity and tissue-exposure problems that constrain *ANO1* modulators are set out in detail [76].

Two further studies bear on how an ANO1-directed intervention would be used (Figure 2a). In head and neck cancer, p90RSK was identified as a mediator of cisplatin resistance and TMEM16A expression as an upstream regulator through the ERK pathway and as a candidate biomarker of response to p90RSK-targeted therapy, with the p90RSK inhibitor BI-D1870 combined with cisplatin proposed on that basis [77]. This is consistent with the combination logic reached independently from the 11q13 analysis, and it places ANO1 in the same category as VRAC: a marker that selects the combination rather than the target of the combination itself.

The second concerns the therapeutic window, which is the objection that applies to every blocker considered here. In mice, the ANO1 inhibitor CaCCinh-A01 suppressed calcium-activated chloride currents, reduced ANO1 protein, alleviated ionising radiation-induced intestinal injury and enhanced the radiosensitivity of colorectal cancer cells and xenografts, with the protective effect attributed to NKCC1 upregulation, raised intracellular chloride and inhibition of p53-mediated DNA damage signalling in intestinal epithelium [78]. If that dissociation holds, ANO1 blockade would widen rather than narrow the therapeutic window during radiotherapy, which is the opposite of the situation with LRRC8 activation (Section 5.1). The result rests on one laboratory and on a tool compound whose selectivity is not established to the standard of Section 7, so it is reported as a direction worth testing rather than as an established property.

#### 5.4.5. Structure-Guided Pharmacology

*ANO1* is the only channel in this review for which distinct inhibitor pockets have been visualised. The pore blocker 1PBC occupies a pocket external to the extracellular neck and blocks conduction while the channel remains open-like [79], whereas tamsulosin wedges into an allosteric pocket in the extracellular domain and stabilises a closed, preopen conformation [80]. Structure-based design therefore has two concrete targets rather than one hypothetical one [32].

### 5.5. CFTR: Human Genetics, and the Only Channel with an Approved Activator

*CFTR* is, alongside VRAC, the second channel here for which the therapeutic logic is restoration rather than blockade, and, unlike VRAC, it has a human genetic natural experiment. Cystic fibrosis has been categorised as a familial colorectal cancer syndrome, with screening beginning at age 40 and at age 30 in transplant recipients, and the risk extends to heterozygous carriers of *CFTR* variants; reduced *CFTR* expression has also been observed in sporadic colorectal cancer and is associated with poorer survival [81]. In a matched case-control study, cystic fibrosis was associated with increased risk of colorectal cancer (odds ratio 10.0, 95% confidence interval 1.2–85.6), adenomas (odds ratio 3.34, 95% confidence interval 1.23–9.08) and advanced adenomas (odds ratio 6.95, 95% confidence interval 2.30–21.01) [82]. An extended series from the same centre reported neoplastic lesions in 51.8% of patients versus 24.1% of matched controls (odds ratio 3.41, 95% confidence interval 2.32–5.02) and colorectal cancer in 4.9% versus 0.8% (odds ratio 6.26, 95% confidence interval 1.39–28.26), with a mean age at cancer diagnosis of 40.5 years [83]. The two studies share a centre and investigators and are therefore an extension rather than an independent replication. Moreover, both are retrospective analyses of patients who underwent colonoscopy, so selection bias is not excluded; the very wide confidence interval on the first cancer estimate reflects five events against one.

Mouse genetics supplies the causal direction that epidemiology cannot. *Cftr* was identified as a candidate colorectal cancer driver in Sleeping Beauty transposon screens, and intestinal-specific *Cftr* knockout in *ApcMin* mice produced significantly more tumours in colon and small intestine; decisively, in *Apc* wild-type mice *Cftr*, deficiency alone caused intestinal tumours in over 60% of animals by roughly one year, establishing an independent tumour suppressor rather than a modifier of *Apc*. Colon organoid formation increased in *Cftr*-mutant mice; transcriptomic analysis of the tumours showed dysregulation of Wnt/β-catenin target genes; and, in early-stage human colorectal cancer stratified by recurrence risk, loss of *CFTR* expression was significantly associated with poor disease-free survival [84]. *CFTR* acts as a tumour suppressor in a range of sporadic epithelial cancers, largely through silencing of its expression by epigenetic and other mechanisms [85].

The most recent synthesis proposes that cystic fibrosis be conceptualised as a niche-centric hereditary colorectal cancer predisposition syndrome, in which germline *CFTR* dysfunction chronically destabilises epithelial identity in addition to increasing mutational burden, remodelling stem-cell regulation, promoting hypoxia and oxidative stress, perturbing microbial ecosystems, driving chronic immune activation and stromal remodelling, and inducing epithelial–mesenchymal plasticity. The authors distinguish this from oncofoetal reprogramming and note that the plasticity component requires direct validation in human cystic fibrosis intestinal tissue [86]. This account is a hypothesis rather than an established mechanism, but it is notable for locating the tumour-suppressive role of *CFTR* in epithelial identity maintenance rather than in chloride conductance as such.

The reported association between *CFTR* loss and reduced 5-fluorouracil accumulation does not establish direct permeation of the drug through *CFTR*, and the mechanism should remain classified as indirect or unresolved until isotope-tracer transport, electrophysiological permeation, or transporter-rescue experiments are available. With that caveat, oxidative stress-induced S-glutathionylation of *METTL16* at cysteine 548 has been proposed to drive its degradation and consequent loss of N6-methyladenosine modification, enabling *IGF2BP3* overexpression, which destabilises *CFTR* messenger RNA and reduces 5-fluorouracil uptake; *IGF2BP3* knockdown resensitised resistant cells in vitro and in vivo; high *IGF2BP3* correlated with poor survival across 112 colorectal cancer tissues; and isoliquiritigenin synergised with 5-fluorouracil [87]. The original description of *CFTR* as an ABC transporter is structurally defensible, since *CFTR* is ABCC7, but functionally imprecise: *CFTR* is an ATP-gated Cl^−^/HCO_3_^−^ channel rather than an active transporter. The claim is reported here as the mechanism the authors proposed rather than as an established route of drug entry.

*CFTR* is also the only protein in this review for which approved, target-directed modulator therapies exist. These fall into two mechanistic classes that are often conflated: correctors such as elexacaftor and tezacaftor, which improve folding and trafficking of mutant protein, and potentiators such as ivacaftor, which increase the open probability of channels already at the membrane. Only the latter is an activator in the sense relevant to a gain-of-function anti-cancer rationale, and both classes are licensed for defined *CFTR* dysfunction rather than for wild-type activation. A systematic analysis of expression signatures for 48 human ABC transporter genes across 8562 patients and 14 cancer types found that low *CFTR* predicted reduced survival in lung adenocarcinoma, as confirmed in a cohort of 246 patients with a smoking history and in 143 never-smokers, and *CFTR* potentiators reduced proliferation at therapeutically relevant concentrations in lung adenocarcinoma cells that natively express the channel [88]. This was an exploratory finding within a discovery-wide analysis rather than a prespecified hypothesis, and no animal efficacy data accompany it. A further caution applies specifically to repurposing the approved modulators: transcriptomic profiling of cystic fibrosis airway epithelial cells exposed to elexacaftor/tezacaftor/ivacaftor showed increased defensin expression, decreased MMP10, MMP12 and heme oxygenase expression, and pathway-level reductions in inflammatory signalling, cell proliferation and MHC class II antigen presentation [89]. Therefore, the antiproliferative effect of these agents has not been separated from channel-independent pleiotropy. The genetic-restoration control that would separate them has not been reported, and this is the principal deficit in the *CFTR* repurposing case.

Two studies published since the original search supply part of the missing evidence (Figure 2b). In osimertinib-resistant EGFR-mutant non-small-cell lung cancer models, a drug-library screen identified the *CFTR* potentiator ivacaftor as synergistic with osimertinib in vitro and in vivo, with the proposed mechanism being ivacaftor-driven colocalisation of *CFTR* and PTEN at the plasma membrane, enhanced PTEN function and consequent inhibition of PI3K/AKT signalling [90]. This provides animal-efficacy evidence for a *CFTR* potentiator in an oncology setting and it is directionally consistent with the restoration logic argued here. The proposed mechanism is nonetheless a scaffolding effect on PTEN localisation rather than an effect of anion conduction, and no comparison with genetic restoration was reported, so the study does not establish that chloride flux is the operative variable.

The second study does make that comparison. In hepatocellular carcinoma, *CFTR* expression and function correlated inversely with progression, and *CFTR* activation by genetic and by pharmacological means reduced intracellular chloride and calcium, suppressed RUNX1 and NF-κB, altered CSF-1, TGF-β, CCL20 and CCL22 secretion, shifted macrophage polarisation toward the M1 phenotype, increased M1 macrophage and CD8+ T-cell infiltration, reduced regulatory T-cell proliferation and infiltration, and enhanced the efficacy of the PD-L1 blockade [91]. Running the genetic and the pharmacological arm in parallel is the control identified above as missing for *CFTR* potentiators, and the concordance of the two arms is what allows the effect to be attributed to *CFTR* function rather than to channel-independent pleiotropy of the modulators. Two qualifications follow. The mechanism is immunological and operates through altered ion homeostasis and transcription-factor activity rather than through drug permeation. Therefore, it is a third mechanistic route for *CFTR* rather than an extension of the 5-fluorouracil account above. Furthermore, hepatocellular carcinoma is a third tissue, which neither confirms nor contradicts the epithelium-versus-glioma split but does show that the direction in liver follows the epithelial rather than the glial pattern.

A second tissue points in the same direction as glioma, albeit with weaker evidence. In prostate cancer, *CFTR* expression was reported to be increased in chemoresistant tumour tissue and in the cisplatin-resistant LNCaP/CP line relative to parental cells; cisplatin reduced *CFTR* expression in a concentration-dependent manner, and *CFTR* silencing increased the loss of viability produced by cisplatin, with autophagy inhibition reproducing that effect and proposed as the intermediate step [92]. The evidence is weaker in kind than either the intestinal or the glial case, resting on a single resistant subline with no animal arm, and the endpoint is acquired chemoresistance rather than tumour initiation, which is a different question from the tumour-suppressor claim in intestine. It matters for the directional argument all the same because the prostate comprises differentiated secretory epithelium, and the epithelial-state interpretation offered below would not predict it.

Finally, the direction of effect reverses within *CFTR* itself. In malignant glioma, suppression of *CFTR* channel function or knockdown reduced cell viability, whereas overexpression increased it; overexpression suppressed apoptosis and promoted progression in subcutaneous and orthotopic xenografts through an Akt/Bcl2 pathway, with inhibition of PI3K/Akt abolishing the associated Bcl2 upregulation, and *CFTR* protein was significantly increased in glioblastoma patient samples [93]. The evidence on the epithelial side is stronger in kind, resting on conditional knockout and human genetics, whereas the glial evidence rests on cell lines and xenografts. However, both directions were reproduced by genetic manipulation and the proposed mechanisms differ: Wnt and stem-cell regulation versus Akt/Bcl2. The most parsimonious reading of the epithelium-versus-glia contrast is that the tumour-suppressive role of CFTR is tied to the differentiated secretory epithelial state, which is absent in glia. That reading is a hypothesis generated here, which has not been tested directly, and the prostate result above is not consistent with it in its simplest form, since prostate contains secretory epithelium. An equally available and equally untested alternative is that the two sets of observations measure different things, tumour initiation in the intestinal case and acquired chemoresistance in the prostate and glial ones, in which case tissue is not the only variable that has to be indexed. The experiment that would discriminate is a side-by-side comparison of lineage-specific conditional Cftr deletion in epithelial and glial compartments on a common genetic background, with tumour initiation and platinum response measured as separate endpoints.

### 5.6. ANO6/TMEM16F: Direction Unresolved

An apparent contradiction in the *ANO6* literature can be resolved, on inspection, as a mismatch between the measurement entities. The claim of a tumour-promoting role in breast cancer derives from exon-based clustering of murine breast tumour transcriptomes, in which alternative exon usage rather than total transcript abundance was associated with metastasis [94]. The opposing claim, that low *ANO6* predicts poor outcome, derives from human bioinformatic analyses of total transcript abundance, and the review reporting it states that the prognostic effect in breast cancer has not been validated at the experimental level [95]. The most recent comprehensive treatment of TMEM16F biology restates the structural plasticity underlying its dual function without resolving the oncological direction [96]. *ANO6* is therefore retained here as an area of unresolved evidence rather than as a supporting example in either direction. The one experimentally derived directional result is in gastrointestinal stromal tumour, where *ANO6* was downregulated and its restoration induced apoptosis, pyroptosis and ferroptosis [97]; the wider frame is provided by analyses of anoctamin contributions to cell survival and death [2].

### 5.7. Bestrophin-1: Evidence Insufficient to Establish a Direction

Canonical VRAC is formed by LRRC8 heteromers, and the following observation should not be read as contradicting that. In metastatic oral squamous cell carcinoma cells, knockdown of both TMEM16A and *LRRC8A* attenuated the volume-regulated current while *BEST1* knockdown affected proliferation, and a proximity ligation assay showed TMEM16A and *LRRC8A* co-localising under isotonic but not hypotonic conditions [98]. Rather than showing that VRAC has multiple molecular identities, this suggests that the volume-sensitive current measured in that particular line may contain contributions from additional conductances or interacting proteins. It is a single cell line, and *BEST1* is retained here as a declared gap rather than as a conclusion. The observation nonetheless qualifies the LRRC8-centred account of Section 4.1 and should be read alongside it. Accordingly, the available BEST1 evidence concerns proliferation and current composition rather than chemotherapy or radiotherapy response, so no treatment-direction inference is made.

### 5.8. ANO7 and ANO9: Stratification Rather than Blockade or Restoration

Two scramblase-subgroup members illustrate what happens when a protein has cancer relevance but no druggable pocket.

*ANO7* is expressed almost exclusively in prostate tissue. Its expression is inversely correlated with the Gleason score and pathological stage [99], and circulating autoantibodies against it are detectable at higher rates in prostate cancer patients than in controls, suggesting diagnostic utility from peripheral blood [100]. Human genetic evidence has since strengthened considerably: a meta-analysis of ten genome-wide association studies comprising 19,378 cases and 61,620 controls of African ancestry identified an African-specific stop-gain variant in *ANO7* among nine novel susceptibility loci [101]. The immunotherapeutic rationale rests on direct experimental evidence: T-cell lines raised against an HLA-A2-restricted epitope specifically lysed HLA-A2- and *ANO7*-expressing tumour cells, and *ANO7*-specific T-cell frequency rose in patients following a prostate-specific antigen-based vaccine [102]. A comprehensive review concluded that restricted tissue expression and demonstrated immunogenicity make *ANO7* a candidate for antibody- or T-cell-based immunotherapy while cautioning that its channel-versus-scramblase identity remains unsettled [103].

Two 2025 studies moved *ANO7* from correlation toward function, and both point away from conduction. RNA sequencing of prostate cells engineered to express *ANO7*, combined with single-cell data, targeted metabolomics, lipidomics and mitochondrial imaging, showed downregulation of MYC-pathway and oxidative phosphorylation genes, a metabolic shift toward glycolysis, reduced aspartate, altered mitochondrial morphology, and lipid remodelling toward longer and more unsaturated acyl chains [104]. Spatial transcriptomics of prostatectomy specimens found a distinct on-off epithelial expression pattern, with *ANO7*-positive epithelium enriched for luminal cells and *ANO7*-negative regions showing inflammation and features of proliferative inflammatory atrophy. The co-expression signature was enriched for androgen-receptor-regulated genes and arachidonic acid metabolism, and in low-grade disease it correlated with a better response to therapy [105]. That last observation matters here: it converts *ANO7* from a pure antigen into a candidate predictor of treatment response, which is the category considered in this review. Neither study, however, tested channel or scramblase activity, so the metabolic phenotype cannot presently be assigned to ion or lipid movement.

*ANO9*/TMEM16J behaves in opposite directions in different gastrointestinal cancers. In colorectal carcinoma, reduced expression was associated with tumorigenesis and poor prognostic outcome [106]. In oesophageal squamous cell carcinoma, the relationship reverses: depletion reduced proliferation, invasion and migration and increased apoptosis through centrosome-related gene regulation, and high expression was associated with significantly worse survival [107,108]. In pancreatic cancer, *ANO9* again behaves as a conventional oncogene, associating with EGFR to drive proliferation, with knockdown augmenting the effect of EGFR inhibitors and cytotoxic agents and high expression independently predicting poor prognosis [109].

Neither protein has a modulator. Both nonetheless bear on treatment decisions—*ANO7* as an antigen and serum marker and *ANO9* as a predictor of response to anti-EGFR therapy. They mark a third category in the framework presented in Section 6: neither block nor restore, but stratify.

## 6. The Directionality Problem

Grouping these structurally unrelated proteins as a single therapeutic category generates immediate contradictions. Loss of *LRRC8A* or *LRRC8D* confers platinum resistance, so the intervention implied is preservation, activation or use as a predictive biomarker. Loss of *CFTR* removes a tumour suppressor in intestinal and lung epithelium and is associated with 5-fluorouracil resistance, so the intervention implied is restoration. Gain of *ANO1*, ClC-3 or CLIC1 confers resistance, so the intervention implied is blockade. And CFTR reverses the direction between epithelium and glia, so that a single protein appears on both sides of the ledger depending on tissue. A prostate report of CFTR-dependent cisplatin resistance points in the same direction as the glial evidence, on a chemoresistance rather than a tumour-initiation endpoint (Section 5.5). The LRRC8 entry carries its own qualification, since the preserve-or-activate direction is established for platinum uptake and contrary reports exist for non-platinum agents (Section 5.1). Table 1 sets this out; Figure 3 shows it.

**Figure 3:**
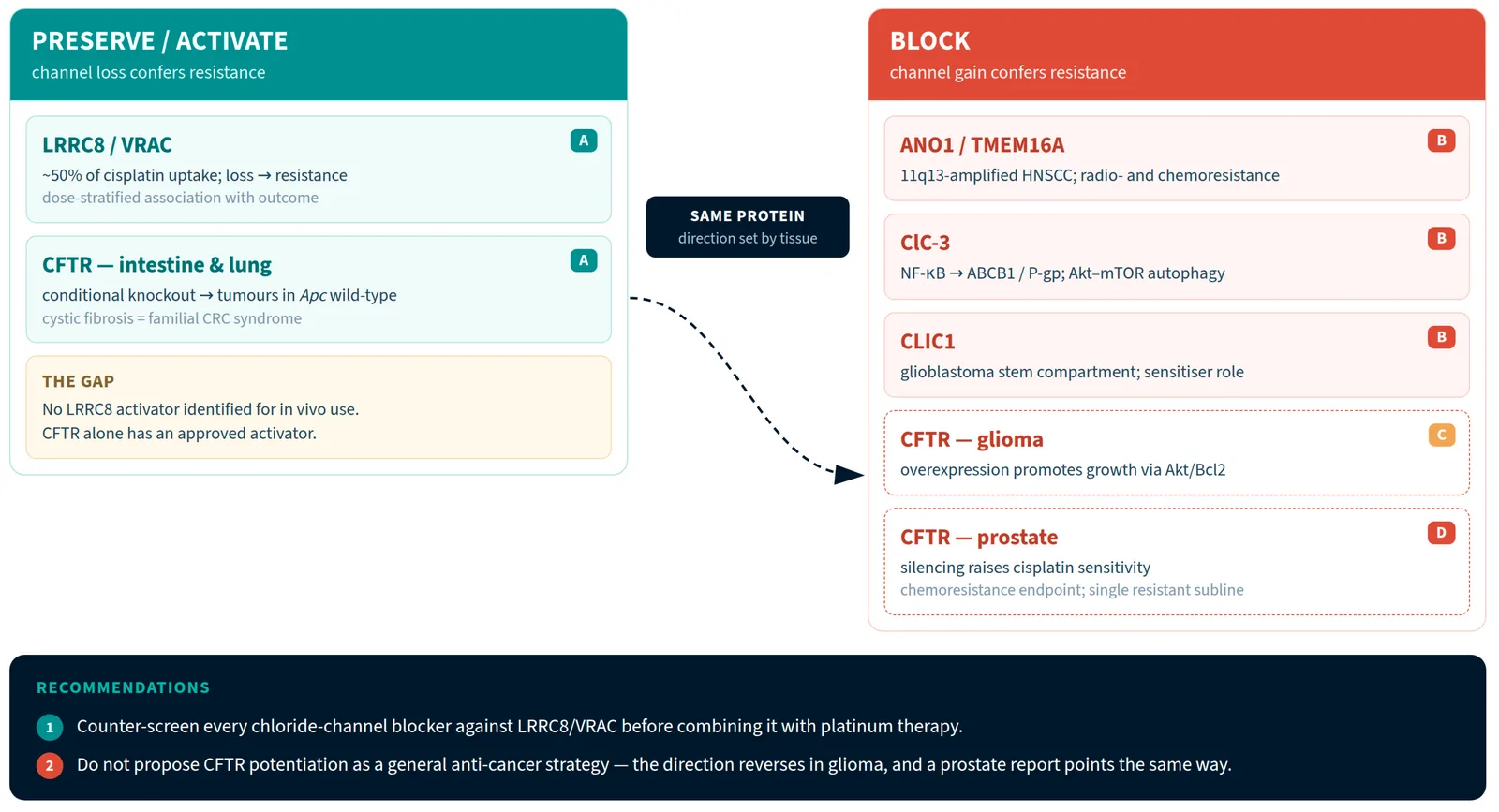
The therapeutic direction reverses between channels and within one channel. Left, channel loss confers resistance and the intervention implied is preservation, activation or biomarker-guided dosing. Right, channel gain confers resistance and the intervention implied is blockade. CFTR appears on both sides because its direction of effect differs between intestinal or lung epithelium and glioma or prostate; this is the point of the figure rather than an inconsistency. Grades follow the standard of Section 7. CRC, colorectal cancer; HNSCC, head and neck squamous cell carcinoma. The letters A–D on each entry are the target-attribution classes defined in Section 7; their application to each claim is given in Table 2.

**Table 1:** Master summary of chloride channels by tissue context, direction of resistance and evidence grade. Grades follow the standard defined in Section 7 and are assigned per claim rather than per channel.

Channel	Tissue Context	Direction of Resistance	Implied Intervention	Grade
LRRC8A/LRRC8D (VRAC)	HNSCC; breast (BRCA1; p53)	Channel loss → platinum resistance	Preserve or activate; predictive biomarker	A
LRRC8A (VRAC)	Ovarian; colon; breast (non-platinum agents)	Direction not consistent; channel retained or gained in resistant derivatives	None supported outside platinum	D
CFTR	Intestine; lung epithelium	Channel loss → tumour suppression lost; 5-FU resistance	Restore or potentiate	A/C
CFTR	Hepatocellular carcinoma	Low expression/function → progression; reduced checkpoint-blockade efficacy	Restore or potentiate (immune-mediated)	B
CFTR	Glioma	Channel gain → progression	Block (opposite to same protein in epithelium)	C
CFTR	Prostate	Channel gain → cisplatin resistance (chemoresistance endpoint)	Block (opposite to intestinal or lung epithelium)	D
ANO1/TMEM16A	11q13-amplified HNSCC	Channel gain → radio- and chemoresistance	Block	B
ClC-3	NSCLC; breast; cervical	Channel gain → P-gp; autophagy	Block	B/C
CLIC1	Glioblastoma	Channel gain → stem compartment	Block (as sensitiser)	B
ANO6/TMEM16F	Direction disputed	Unresolved	None supported	D
Bestrophin-1	Oral SCC (single line)	No directional evidence identified	None supported	E

HNSCC, head and neck squamous cell carcinoma; NSCLC, non-small-cell lung cancer; SCC, squamous cell carcinoma; 5-FU, 5-fluorouracil; P-gp, P-glycoprotein; VRAC, volume-regulated anion channel.

**Table 2:** Target-attribution classes with source traceability. Each claim is assessed against the criteria described in Section 7. Classes A–E are defined by this review for target attribution and are not GRADE certainty ratings. The final column records separately whether any experiment distinguishes dependence on the protein from dependence on anion transport through it—a conduction-deficient rescue, pore mutant, or equivalent separation-of-function design.

Claim	Genetic Loss Reproduces?	Structural Engagement?	Independent Replication?	Class	Limiting Factor	Transport Causality Tested?
VRAC mediates platinum uptake	Yes (subunit-specific)	Yes	Yes	A	—	Partial—subunit-dependent permeability; pore mutants alter selectivity
VRAC loss confers cisplatin resistance in HNSCC	Yes (CRISPR knockout; rescued by re-expression)	—	Yes	A	—	No
LRRC8A direction outside platinum agents	No	—	No	D	Conflicting reports; expression-based designs; no conduction-deficient control	No
CFTR suppresses intestinal tumours	Yes (Apc wild-type alone)	—	Yes	A	—	No
ANO1 drives treatment resistance	Yes (dissected from CCND1)	Yes (two pockets)	No	B	Replication	No
ANO1 blockade widens the radiotherapy window	No	—	No	D	Tool-compound selectivity unverified	No
CLIC1 sustains GBM stem cells	Yes (shRNA and antibody)	Yes (R29 mutant)	No	B	Replication	Partial—antibody-mediated current block reproduces phenotype
CLIC1 organises OSMR/EGFRvIII signalling	Yes (genetic deletion)	—	No	B	Same collaborating network; no structural engagement	Yes—tmCLIC1 current required
ClC-3 upregulates P-glycoprotein	Yes (bidirectional; transgenic)	—	No	B	Structure; replication	No
CFTR loss confers 5-FU resistance	Yes (IGF2BP3)	—	No	C	Uptake mechanism unverified	No
CFTR supports cisplatin resistance in prostate	Yes (siRNA)	—	No	D	Single resistant subline; no in vivo arm	No
CFTR activation is antitumour	Yes (genetic and pharmacological arms concordant)	—	No	B	Structural engagement; independent replication	Partial—intracellular Cl^−^ and Ca^2+^ measured
Ivacaftor synergises with osimertinib	No	—	No	C	Proposed mechanism is PTEN scaffolding, not conduction	No
ClC-3 autophagy sensitises to cisplatin	Yes (knockdown, two tumour types)	—	Yes	C	Tool selectivity in the original in vivo arm; no conduction-deficient control	No
ClC-3 sequesters drug in organelles	No (gain-of-function only)	—	No	C	Loss-of-function untested; drug used is not a weak base	No
ANO6 direction in breast cancer	No (bioinformatic)	—	No	D	No experimental validation	No
Lig8 modulates ClC-3	No (no experiment)	No (docking pose)	No	Not gradable	No experimental data	No
Bestrophin-1 in cancer	No	—	No	E	No directional evidence identified; single-line current composition study	No

GBM, glioblastoma; 5-FU, 5-fluorouracil; shRNA, short hairpin RNA. Transport causality was judged as tested only where a conduction-deficient or pore-mutant construct, or an equivalent separation-of-function design, was reported.

Two conclusions follow. The first is that chloride channel blocker is an oncologically hazardous abstraction, since it names a presumed activity rather than a molecular class, because a compound that inhibits VRAC could in principle reduce platinum uptake and cause resistance rather than relieve it. This is not a theoretical concern. Three separate agents are now documented to act on more than one of these proteins: niclosamide on *ANO1* and *ANO6* [110]; luteolin on *ANO1* and CLIC1 [54]; and DCPIB across a range of unrelated targets, as discussed in Section 7. None of the three has been counter-screened against LRRC8/VRAC. In contrast, Ani9 is the only compound in this review for which a lack of inhibition of VRAC, ANO2, *CFTR* and ENaC has been reported, at a concentration that fully blocks its intended target; this constitutes a limited counter-panel rather than a demonstration of global selectivity [111].

The second conclusion is that the reversal occurs within a single channel across tissues as well as between channels, so recommendations must be indexed to channel and tissue jointly rather than to channel alone. This is not a refinement of the first point but a distinct point, and it is the reason the framework of this review is organised as a matrix rather than as a list.

Two recommendations follow directly. Any chloride-channel modulator advanced toward an oncology indication should be counter-screened against LRRC8/VRAC before combination with platinum-based therapy; we did not identify a case in which this counter-screen has been performed systematically. The counter-screen is specified for platinum combinations because that is where the uptake evidence lies, not because VRAC is irrelevant elsewhere. And CFTR potentiation should not be proposed as a general anti-cancer strategy, because the direction of effect reverses in glioma, a prostate report points in the same direction, and the agents concerned are already approved and in wide use. The case for the second recommendation has strengthened rather than weakened during revision: CFTR potentiation now carries in vivo antitumour efficacy data in two tissues (Section 5.5), which makes the absence of any glioma-directed safety assessment an immediate rather than a theoretical concern.

## 7. The Tool-Compound Problem and a Proposed Evidence Standard

### 7.1. Off-Target Actions of the Most Widely Used Blocker

Much of this literature rests on tool compounds, and the most widely used of them is not selective. DCPIB, commonly described as the most selective available blocker of the volume-regulated anion channel, has been reported across five independent peer-reviewed studies to inhibit mitochondrial respiration in a channel-independent manner [112], to inhibit several glutamate transport pathways [113], to block inwardly rectifying potassium channels with weak phosphatidylinositol 4,5-bisphosphate affinity [114], to activate TREK potassium channels [115], and to gate large-conductance calcium-activated potassium channels directly while raising intracellular calcium [116]. Each was reported by a different group. On the published record, the compound most often called selective is promiscuous.

A general principle explains why this recurs. Ion-channel modulators are frequently lipophilic, amphiphilic or weakly basic, properties that aid membrane access but increase plasma protein binding, tissue accumulation and non-specific membrane interaction. Therefore, that apparent potency must be interpreted against unbound rather than nominal exposure [8]. One practical consequence governs how the potencies tabulated in Section 8 should be read: a half-maximal value obtained in serum-containing medium and one obtained under serum-free conditions are not the same quantity, because the unbound fraction differs between them. As such, a report that does not state which condition was used leaves its absolute value uninterpretable even when the assay itself is sound. Figure 4 summarises the peer-reviewed off-target liabilities that motivate the counter-screening standard.

### 7.2. Selectivity Is Achievable, and Discovery Order Determines the Evidence

There is nonetheless reason to think the problem is tractable rather than intrinsic. Iodinated derivatives of gallic and ferulic acid discriminate between chloride conductances in opposite directions: iodogallic acid inhibited the volume-regulated anion channel with a half-maximal inhibitory concentration near 10 µM in a reversible and weakly voltage-dependent manner while leaving cyclic AMP-activated currents unaffected. Iodoferulic acid, on the other hand, did not affect the volume-regulated current but produced a small, statistically significant and reversible inhibition of cyclic AMP-activated currents [117]. Selectivity across chloride channel families is therefore achievable, which removes the most common objection to the counter-screening recommendation in Section 6.

Discovery methodology matters here as much as structure. Ani9 emerged from a screen of 54,400 compounds and inhibits *ANO1* with a half-maximal inhibitory concentration of 77 ± 1.1 nM, more than eighteen-fold more potent than T16Ainh-A01 and MONNA when measured in the same assay, with no detectable effect on ANO2, *CFTR*, VRAC or ENaC at 1 µM in the limited panel tested [111]. Translating such hits into structure–activity relationships required automated patch-clamp platforms engineered to prevent the channel’s characteristic rundown during cumulative concentration–response testing [118]. The two-tier logic—optical high-throughput screening followed by direct electrophysiological confirmation—is what we have argued should govern ion-channel programmes generally [8].

Niclosamide illustrates the opposite discovery order. It was identified as an inhibitor of SARS-CoV-2 spike-induced syncytium formation in a phenotypic screen [119], and only subsequently was its activity against both *ANO1* and *ANO6* characterised [110], with cryo-EM later locating a drug-binding pocket within the TMEM16F scrambling groove [120]. Its cross-reactivity between anoctamin paralogues is documented rather than inferred, which is why it fails the counter-screening standard proposed in Section 6. The same literature also contains an unresolved disagreement worth stating plainly. Six avermectin analogues were reported to inhibit TMEM16A-mediated currents with half-maximal inhibitory concentrations of 0.15 to 1.32 µM and maximal inhibition of 65 to 87% by whole-cell patch clamp, with ivermectin and avermectin B1 suppressing xenograft growth, while structurally related macrolides without TMEM16A activity did not [121]. The study that characterised niclosamide reached the opposite conclusion for ivermectin specifically [110]. The two used different assay formats and cell backgrounds, and we do not attempt to adjudicate between them here; the disagreement is noted because ivermectin is widely available and its status as an *ANO1* modulator should not be treated as settled.

A selectivity problem created by small molecules need not be solved with small molecules. The monoclonal antibody directed at transmembrane CLIC1 described in Section 5.2 achieves target restriction by molecular recognition rather than by pharmacophore optimisation [53], and the same approach applies in principle to any protein in this set carrying an accessible extracellular epitope. It does not remove the counter-screening requirement, because an antibody raised against one conformation of a metamorphic protein may not distinguish it from the same protein in another state, but it does mean that the poor selectivity of the available small molecules is a property of the chemical matter rather than of the target class.

### 7.3. A Standard Based on Genetic Loss

We therefore grade every claim in this review by whether the phenotype attributed to a compound is reproduced when the protein is removed genetically. Classes A to E below are defined by this review for the specific purpose of target attribution; they are not equivalent to GRADE certainty ratings and should not be read as such. Class A requires genetic loss of function, target engagement established at structural resolution, and independent laboratory replication. Class B requires genetic loss with concordant compound data from a single laboratory. Class C covers genetic evidence alone, or a counter-profiled compound alone. Class D covers tool-compound evidence with unverified selectivity, and Class E expression correlation alone. The scheme is set out in Figure 5 and its application to each claim in Table 2.

A second and independent axis must be tracked alongside it, because the classes above cannot capture it. Genetic deletion establishes that a phenotype depends on the protein; it does not establish that the phenotype depends on anion transport through that protein. Every protein considered here has functions that could in principle operate without conduction: LRRC8 complexes carry organic osmolytes and signalling molecules as well as chloride; TMEM16 proteins scramble lipids; CLIC1 exists in a soluble form with reported redox and enzymatic activity; ClC-3 exchanges rather than conducts; and *CFTR* regulates other transporters and organises membrane protein complexes. Distinguishing protein dependence from transport dependence requires a conduction-deficient or scrambling-deficient rescue construct, a pore mutant, or an equivalent separation-of-function experiment. Table 2 therefore records, for each claim, whether such an experiment has been reported. The answer is almost uniformly no, and this is the single largest unaddressed weakness in the field rather than a criticism of any individual study. It rests on two foundations: the established requirement that acute channel inhibition and protein-level remodelling be assessed by separate assays with separate controls and normalisation, since they can produce similar phenotypes while demanding different medicinal-chemistry decisions [8]; and the demonstrated failure of that requirement in this specific literature. It complements rather than replaces GRADE and SYRCLE, neither of which evaluates whether an intervention engaged its intended target—the dominant failure mode in ion-channel pharmacology.

## 8. Reported Modulators and Translational Constraints

Sixteen reported modulators recur in this literature, and Table 3 compares them by reported potency, assay format, selectivity/counter-screening evidence and clinical status. Off-target liabilities and model context are discussed in the accompanying text, where relevant. Values that could not be recovered from the accessible primary text are marked as such rather than estimated, and potencies obtained in incompatible assay formats are not compared. Figure 6 plots only those values recovered exactly.

Three observations emerge from the table. First, the compound with the best-documented selectivity, Ani9, is a research tool with no oncology development programme, while the compounds with clinical development potential are approved agents whose selectivity against other chloride channels is largely unreported. Second, the single most conspicuous absence is a pharmacological activator of LRRC8/VRAC, which Section 5.1 identifies as the intervention that the strongest evidence in this review actually implies; patent databases were not searched, so this is an absence in the published literature rather than a demonstrated absence of such an activator. Third, no registered oncology trial of a chloride-channel modulator was identified in this search, and ClinicalTrials.gov was not systematically searched, so this too should be read as a limitation rather than a finding.

Two ANO1 inhibitors reported since the original search illustrate the same pattern. Hemin emerged from a screen of 2978 approved and investigational drugs with a half-maximal inhibitory concentration of 0.45 µM, no significant effect on intracellular calcium signalling or on CFTR, and only weak inhibition of ANO2 at a concentration that fully blocks ANO1. It also reduced ANO1 protein levels, so its cellular effect combines acute channel inhibition with protein-level loss and the two were not separated [122]. Sophoricoside, a natural isoflavone, inhibited the TMEM16A current with a half-maximal inhibitory concentration of 14.8 ± 1.2 µM and reduced xenograft growth, with its binding site predicted by molecular dynamics rather than resolved experimentally [123]. Hemin is counter-screened more carefully than most entries in Table 3 and sophoricoside is not; neither has an oncology development programme, so the asymmetry described above is unchanged.

Tamsulosin illustrates the discipline the table is meant to impose. It binds a defined allosteric pocket in the extracellular domain of TMEM16A resolved at 2.93 Å with mutagenesis confirming the site, inhibits the current with a half-maximal inhibitory concentration of 7.22 ± 2.00 µM, and shows in vivo efficacy in a mouse model of bone loss [80]. It is an approved oral drug with a known safety profile and a structurally defined interaction with a channel that this review implicates in treatment resistance. It has no oncology efficacy data whatsoever, and transferring the bone-loss result into an anti-cancer rationale would be exactly the kind of inference this review is written to discourage.

## 9. Limitations

Several constraints affect how this review should be interpreted. The search was restricted to PubMed. During revision, the strategy was re-executed and supplemented by controlled-vocabulary searches constructed independently of the original free-text strings; these recovered nine eligible records that the first strategy had missed. A further coverage audit before resubmission recovered fourteen more, including reports that contradict the directions asserted in the submitted version. Two rounds of independently constructed searching therefore both returned eligible records, which indicates that the original free-text strategy under-retrieved substantially rather than marginally, and that the present reference set should be seen as sufficient for the directional argument rather than as exhaustive of the field. Embase, Scopus and Web of Science were not searched, patent databases were not consulted, and ClinicalTrials.gov was not systematically searched. Therefore, statements about the absence of a compound class or a trial are statements about the indexed literature accessible to this search rather than about the field. Bibliographic accuracy was verified programmatically for all cited works, but the full text was examined for only a minority; two intended full-text checks could not be completed because the sources were not deposited in an open repository. One consequence is material: whether the reported potency of triamterene against CLIC1 was obtained under serum-containing or serum-free conditions could not be determined, and, for the reason set out in Section 7.1, those two conditions do not yield the same quantity. As such, the absolute potency is not reported and only the source’s relative comparison is retained. The CLIC1 literature still originates predominantly from one collaborating network, although one of the records added during revision comes from a group outside it (Section 5.2). Negative and unpublished results were inaccessible, so publication bias is uncorrected. Conclusions for *ANO6* and bestrophin-1 are withheld rather than reported in a weak form. The scope is also a selection rather than the universe of chloride-transporting proteins: CLNS1A, for example, has been reported to confer platinum resistance in non-small-cell lung cancer through chloride-channel-dependent efflux, with a conduction-defective mutant losing the effect [124], and it is not treated here because the proteins examined were selected for carrying both directional and pharmacological evidence. Its existence indicates that the directional framework should be expected to extend beyond this set. Finally, the interpretation offered for the reversal of *CFTR* direction between epithelial and glial tumours is a hypothesis generated by this review and has not been tested.

An additional molecular boundary applies to the LRRC8-centred account of the volume-regulated current. *TTYH1* and *TTYH2* have been reported to carry *LRRC8A*-independent volume-regulated anion currents in several cancer cell lines, including SNU-601 gastric cancer cells, in which the current was almost absent from a cisplatin-resistant derivative and was partly restored by a histone deacetylase inhibitor; silencing either gene abolished the current and re-expression restored it [125]. The association with a resistant derivative is recorded because it is consistent with the VRAC account in Section 5.1, but platinum transport through *TTYH1* or *TTYH2* was not measured and no treatment-direction effect was tested. The channel assignment is in any case contested: two independent cryo-electron microscopy studies resolved *TTYH1–3* as dimeric membrane proteins with an extended extracellular domain and no ion-conducting pathway, and reported no *TTYH2*-dependent channel activity in cells [126,127]. That sequence, an asserted conductance followed by a cancer-associated phenotype and then by a structural refutation, is precisely the situation that the standard in Section 7 is intended to handle. These proteins are therefore included here to define a boundary for the molecular interpretation of the volume-regulated current rather than being entered into the graded therapeutic matrix.

## 10. Conclusions

Chloride flux is a real axis of anti-cancer drug response, but it is not a single one, and treating it as a target class would be a mistake with a foreseeable clinical cost. The strongest evidence in this review—*LRRC8A* and *LRRC8D* as determinants of platinum uptake, with dose-stratified prognostic associations reported in patients, and *CFTR* as an intestinal tumour suppressor supported by conditional knockout and human genetics—points toward preservation, activation and biomarker-guided dosing rather than blockade. The evidence for ANO1, ClC-3 and CLIC1 points in the other direction. CFTR points both ways in different tissues. Neither half of this is uniform: the LRRC8 direction is established for platinum uptake and is contradicted for several non-platinum agents, and the CFTR reversal is reported in prostate as well as in glioma. Recommendations must therefore be indexed to channel and tissue jointly.

Two specific actions follow. Any chloride-channel blocker advanced toward an oncology indication should be counter-screened against LRRC8/VRAC before combination with platinum therapy, a step that is technically achievable and has not been systematically performed. And CFTR potentiation should not be advanced as a general anti-cancer strategy given the opposite direction of effect reported in glioma and, on weaker evidence, in prostate. Beyond these, two gaps are consequential. The first is the absence of a pharmacological LRRC8 activator, since the best-supported finding in this literature currently has no therapeutic instrument attached to it. The second is methodological: almost no claim in this field has been tested with a conduction-deficient rescue construct, so the field does not yet know, for any of these proteins, whether the reported cancer phenotypes depend on anion transport at all. Meanwhile, claims in this field should be graded by whether they survive removal of the channel itself, because the compounds used to make them frequently do not act where they are assumed to.

## Figures and Tables

**Figure 1:**
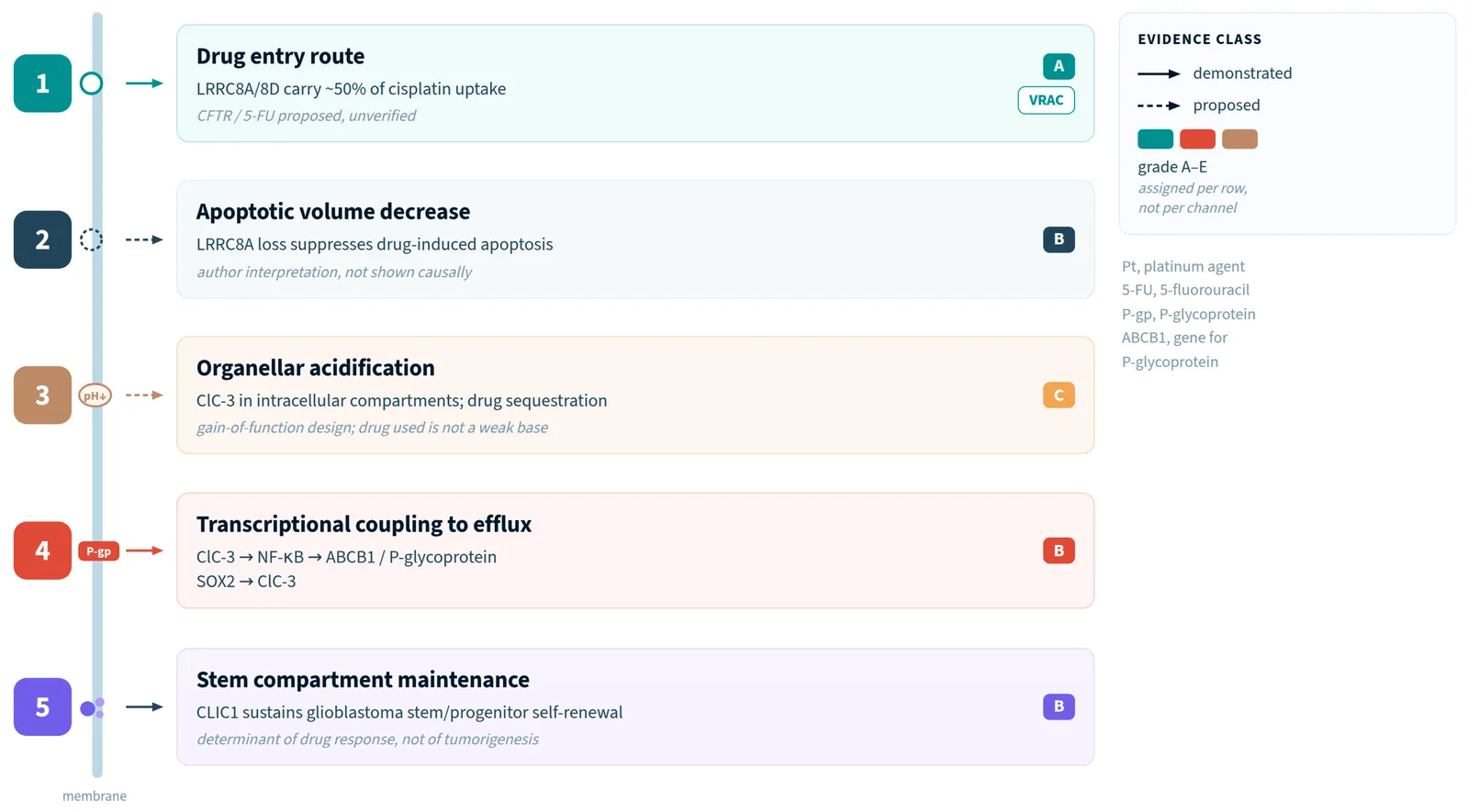
Five routes by which chloride flux alters anti-cancer drug response. Numbered rows correspond to Section 4.1, Section 4.2, Section 4.3, Section 4.4 and Section 4.5. Arrow style encodes evidence class (solid, directly demonstrated; dashed, proposed or indirect) and the coloured tag gives the grade assigned under the standard of Section 7. Grades are assigned per mechanism rather than per channel, because the same protein may be supported at different strengths for different mechanisms.

**Figure 2:**
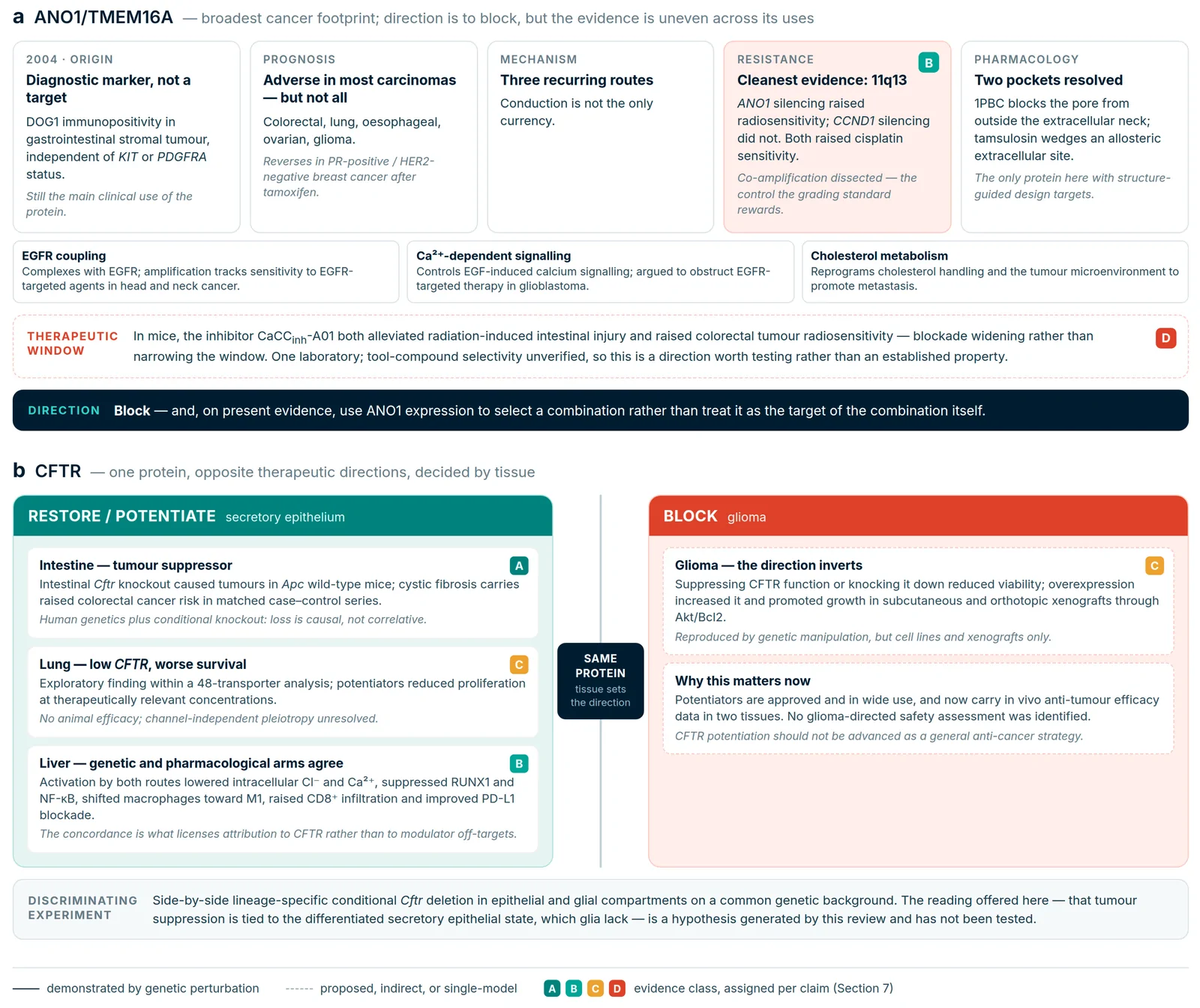
Protein-level summaries for the two proteins carrying the strongest and the most divergent evidence. (**a**) ANO1/TMEM16A: the diagnostic origin, the non-uniform prognostic association, the three recurring mechanistic routes, the resistance evidence dissected from CCND1 co-amplification, the two structurally resolved inhibitor pockets, and the therapeutic-window result that would, if confirmed, distinguish ANO1 blockade from the other blockade targets here. (**b**) CFTR: one protein assigned opposite therapeutic directions in secretory epithelium and in glioma, with the evidence type supporting each direction and the experiment that would discriminate between them. Solid outlines mark relationships demonstrated by genetic perturbation; dashed outlines mark proposed, indirect or single-model relationships. Evidence classes follow the standard of Section 7 and are assigned per claim rather than per protein.

**Figure 4:**
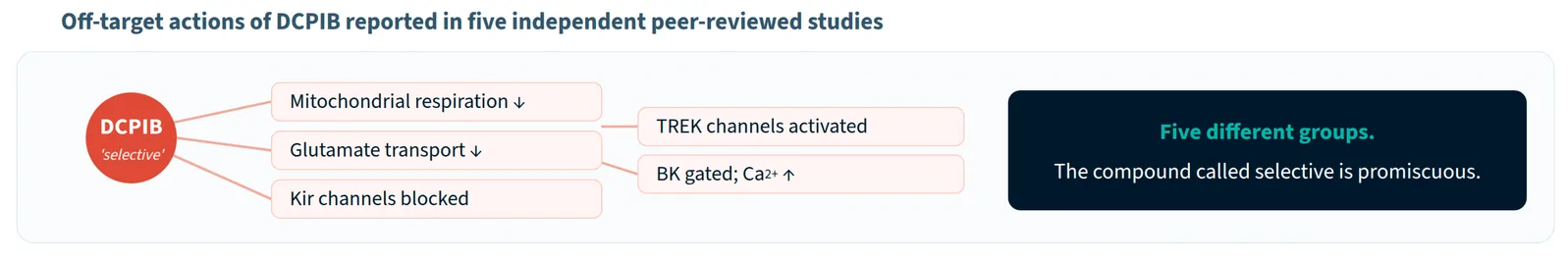
Selectivity problem in the most widely used volume-regulated anion channel blocker. Independent peer-reviewed studies report that DCPIB inhibits mitochondrial respiration, glutamate transport and inwardly rectifying potassium channels, activates TREK channels, and directly gates BK channels while increasing intracellular Ca^2+^. The figure summarises the off-target liabilities discussed in Section 7.1. Connecting lines link DCPIB to each reported off-target action and denote a reported effect rather than a direction of regulation.

**Figure 5:**
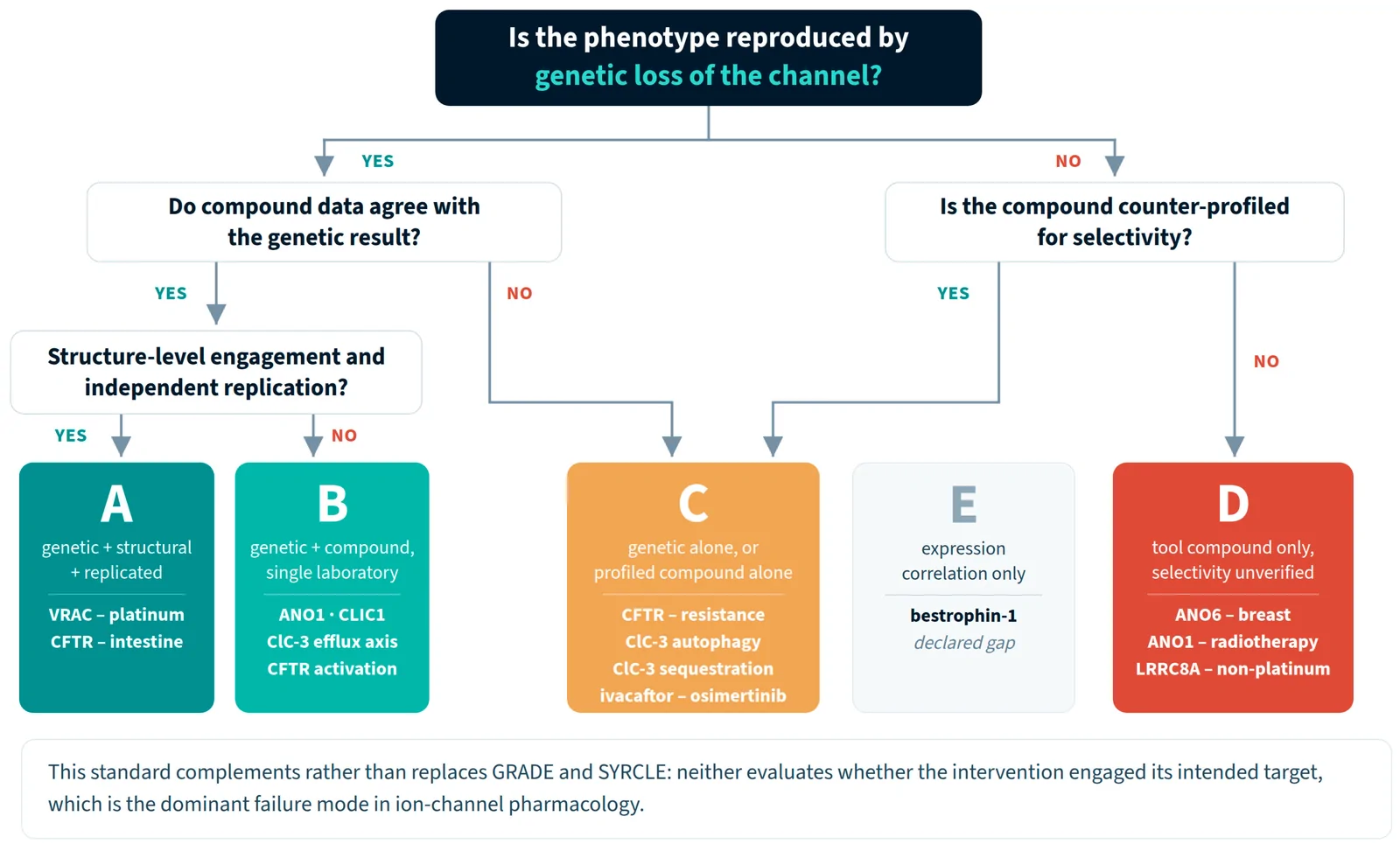
Grading standard applied throughout this review. The first and dominant question is whether the phenotype attributed to a compound is reproduced when the channel is removed genetically; a claim that fails this test cannot rise above Grade C irrespective of the apparent potency of the compound. Terminal nodes show representative claims from Table 2 at each class. The standard complements rather than replaces GRADE and SYRCLE, neither of which evaluates whether an intervention engaged its intended target. Classes A–E are the target-attribution classes defined in Section 7; the criteria for each are shown in the figure and their application to each claim is given in Table 2.

**Figure 6:**
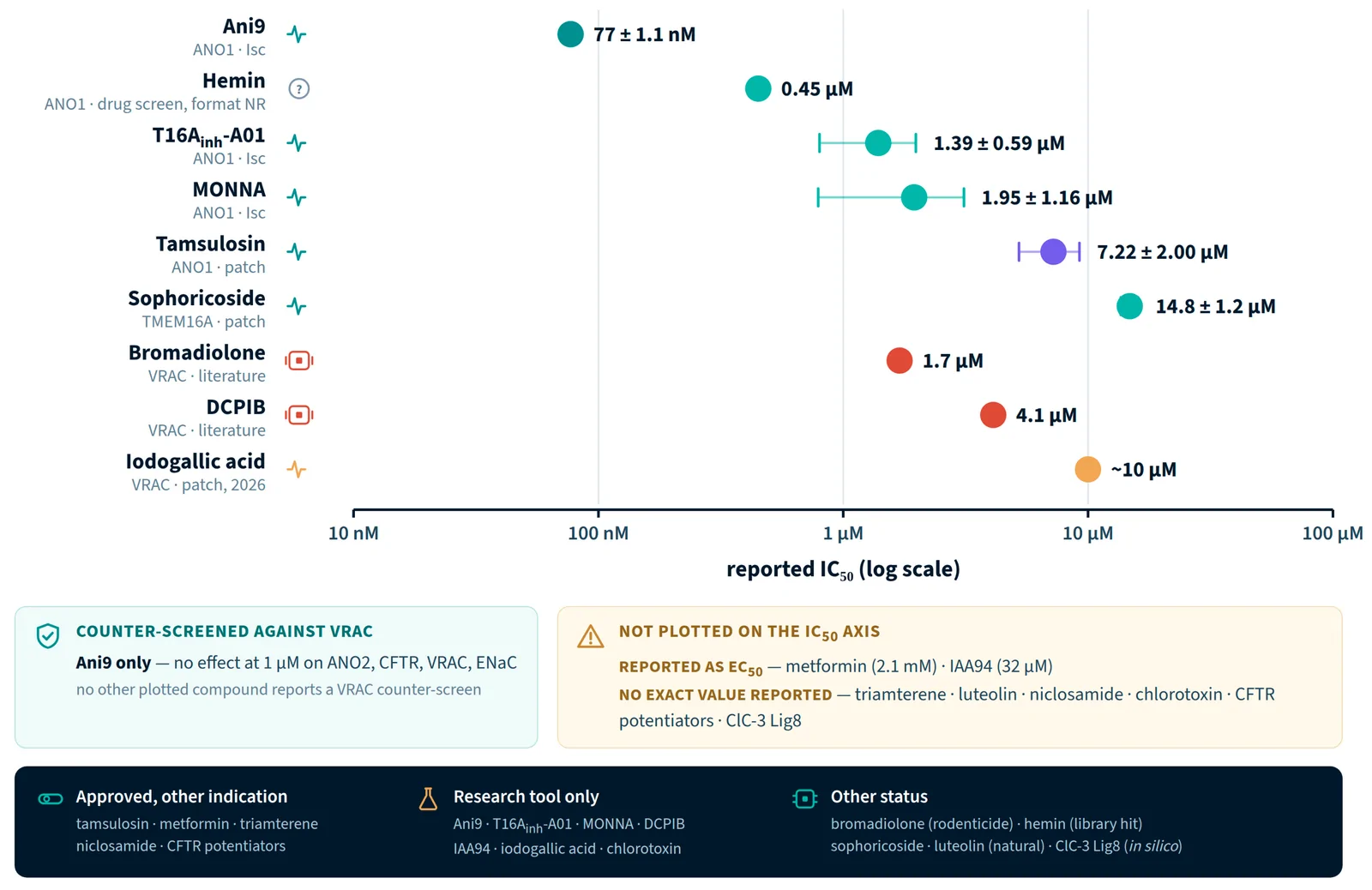
Potency landscape of chloride-channel modulators. Only IC_50_ values are plotted, and only where an exact figure was recoverable from the primary text; assay format differs between rows and values are not mutually comparable. Error bars are standard deviations as reported and are not visible where they fall within the marker. Compounds whose primary source reports an EC_50_ rather than an IC_50_, and compounds for which no exact value was recovered, are listed beside the plot rather than estimated.

**Table 3:** Pharmacological profile of chloride-channel modulators. Potencies obtained in different assay formats are not mutually comparable; the assay is stated for each. Values that could not be recovered from the accessible primary text are marked NR and were not estimated.

Compound	Target (as Claimed)	Reported Potency	Assay Format	Selectivity/Counter-Screen	Clinical Status
Ani9	ANO1	IC_50_ 77 ± 1.1 nM	Apical membrane current, FRT-ANO1	No effect at 1 µM on ANO2, CFTR, VRAC, ENaC	Research tool
T16Ainh-A01	ANO1	IC_50_ 1.39 ± 0.59 µM	Same assay as Ani9	Does not distinguish ANO1 from ANO2	Research tool
MONNA	ANO1	IC_50_ 1.95 ± 1.16 µM	Same assay as Ani9	Does not distinguish ANO1 from ANO2	Research tool
Tamsulosin	ANO1 (allosteric pocket)	IC_50_ 7.22 ± 2.00 µM	Whole-cell patch clamp	Cryo-EM 2.93 Å; R605/E624 mutagenesis	Approved (BPH); no oncology data
Hemin	ANO1	IC_50_ 0.45 µM	Drug screen in PC-3 cells; assay format NR	No significant effect on Ca^2+^ signalling or CFTR; weak ANO2 inhibition at 3 µM	Drug-library hit; no oncology programme identified
Sophoricoside	TMEM16A	IC_50_ 14.8 ± 1.2 µM	Whole-cell patch clamp	NR	Natural product
DCPIB	LRRC8/VRAC	IC_50_ 4.1 µM	Literature value	Kir, TREK, BK, glutamate transport, mitochondria	Research tool
Bromadiolone	LRRC8/VRAC	IC_50_ 1.7 µM	Literature value	NR	Anticoagulant rodenticide; mechanism-based bleeding liability precludes systemic use
Iodogallic acid	LRRC8/VRAC	IC_50_ ~10 µM	Whole-cell patch clamp	No effect on cAMP-activated current	Research tool
Metformin	tmCLIC1 (Arg29)	EC_50_ 2.1 mM	Perforated patch, U87	R29 mutation abolishes effect	Approved (type 2 diabetes)
IAA94	CLIC1	EC_50_ 32 µM	Perforated patch, U87	NR	Research tool
Triamterene	CLIC1	~200-fold lower than metformin; absolute NR	Whole-cell, glioma stem cells	Spares astrocytes, oligodendrocytes, endothelium	Approved (diuretic)
Luteolin	CLIC1 and ANO1	NR	CC-ABPP, SPR; patch clamp for ANO1	Inhibits ANO1; does not inhibit CFTR; VRAC untested	Natural product
Niclosamide	ANO1 and ANO6	NR	—	Cross-reactive between anoctamin paralogues (documented)	Approved (anthelmintic)
CFTR potentiators	CFTR	Therapeutically relevant concentrations; NR	Proliferation, lung adenocarcinoma	Channel-independent pleiotropy partly addressed by concordant genetic activation	Approved (cystic fibrosis)
Chlorotoxin	ClC-3 (as described by authors)	NR	Cervical cancer xenograft	Binds MMP-2 and neuropilin 1 (Kd 0.5–0.7 µM); ClC-3 binding unconfirmed	Research use

IC_50_, half-maximal inhibitory concentration; EC_50_, half-maximal effective concentration; NR, not reported or not recoverable from the accessible text; BPH, benign prostatic hyperplasia; FRT, Fischer rat thyroid; CC-ABPP, cysteine-reactive competitive activity-based protein profiling; SPR, surface plasmon resonance; cAMP, cyclic adenosine monophosphate. No pharmacological activator of LRRC8/VRAC was identified.

## Data Availability

No new data were created or analysed in this study. The reference verification log supporting the bibliographic audit described in Section 2 is provided as Supplementary Table S2.

## Supplementary Materials

The following supporting information can be downloaded at: https://www.mdpi.com/article/10.3390/cancers18203369/s1. Table S1: verbatim search strings for both search rounds, including the controlled-vocabulary searches added in revision, the record counts returned by each, and the operational inclusion and exclusion criteria; Table S2: reference verification log, including DOI-to-PMID resolution and retraction screening for all cited works.

## Abbreviations

The following abbreviations are used in this manuscript:

5-FU	5-fluorouracil
ABC	ATP-binding cassette
ABCB1	ATP-binding cassette subfamily B member 1, encoding P-glycoprotein
ANO	anoctamin
BK	large-conductance calcium-activated potassium channel
BPH	benign prostatic hyperplasia
CFTR	cystic fibrosis transmembrane conductance regulator
CLIC1	chloride intracellular channel 1
CRC	colorectal cancer
DOG1	discovered on GIST-1
EGFR	epidermal growth factor receptor
ENaC	epithelial sodium channel
ETI	elexacaftor/tezacaftor/ivacaftor
GBM	glioblastoma
GIST	gastrointestinal stromal tumour
HNSCC	head and neck squamous cell carcinoma
IC_50_	half-maximal inhibitory concentration
Kir	inward-rectifier potassium channel
LDH	lactate dehydrogenase
P-gp	P-glycoprotein
TMEM16	transmembrane protein 16
VRAC	volume-regulated anion channel
