# Supplementary material for: Chloride Channels as Determinants of Anti-Cancer Drug Response: Why the Therapeutic Direction Reverses Between Channels and Between Tissues
Source: Cancers (Basel). 2026 Oct 18;18(20):3369. doi: 10.3390/cancers18203369 (PMC13702227; doi:10.3390/cancers18203369)
Supplement: Supplementary file 1 [file cancers-18-03369-s001.zip › cancers-4554067-supplementary.pdf]

cancers

MDPI

Supplementary Materials

Chloride Channels as Determinants of Anti-Cancer Drug Response: Why the Therapeutic Direction Reverses Between Channels and Between Tissues

Yohan Seo — Department of Physiology, Dongguk University College of Medicine, Gyeongju, Republic of Korea

Table S1. Search strategy and eligibility criteria

Database: PubMed (NCBI). The original free-text search was executed to 23 August 2026. Backward citation chaining was performed from the reviews retrieved by searches S7, S8 and S12. No lower limit on publication date was applied except where shown in square brackets below. The strategy was re-executed twice after the original search: on 22 September 2026, when every string was re-run and four controlled-vocabulary searches were added (S1.5); and on 28 September 2026, when a coverage audit was run specifically to test the strategy for concept coverage (S1.6). Because PubMed indexing is dynamic, the counts reported below are those available on the stated dates and are not identical to the counts available on 23 August 2026.

S1.1. Search strings executed (verbatim)

```
S1   LRRC8A volume-regulated anion channel cisplatin uptake resistance
S2   CLIC1 glioblastoma proliferation chloride channel
S3   ClC-3 chloride channel drug resistance tumor cells
S4   CFTR tumor suppressor colorectal cancer
S5   CFTR expression chemotherapy resistance cancer cells
S6   cystic fibrosis cancer risk incidence cohort colorectal
S7   (anoctamin OR TMEM16) AND cancer AND review[Publication Type]       [date_from 2024]
S8    chloride channels cancer therapy resistance review                      [date_from
2023]
S9   bestrophin BEST1 cancer proliferation chloride channel
S10  DCPIB LRRC8 VRAC inhibitor selectivity off-target
S11  Ani9 novel potent small-molecule ANO1 inhibitor negligible effect ANO2
S12  (LRRC8 OR VRAC OR CLIC1 OR CLCN3 OR "ClC-3" OR TMEM16A OR ANO1 OR
     ANO6 OR TMEM16F OR CFTR OR bestrophin) AND (cancer OR tumour OR tumor
     OR carcinoma OR glioblastoma)                                            [date_from
2026/02/01]
S13  ANO6 TMEM16F breast cancer prognosis ferroptosis expression
S14  CFTR ovarian cancer invasion promotes
S15  Tamsulosin ameliorates bone loss TMEM16A allosteric site
```

S1.2. Inclusion criteria (operational)

A record was included when all three of the following applied.

(i) It reported a chloride channel or a modulator of one, together with a neoplastic phenotype or a treatment-response endpoint.

(ii) It provided at least one of: genetic perturbation of the channel; direct measurement of current or ion flux; structural data; animal efficacy; or a human clinical correlate.

(iii) English full text or an official abstract was accessible.

S1.3. Exclusion criteria, with recorded reason

Exclusion ground	Applied to
Non-neoplastic phenotype only (monogenic movement disorder, ataxia, muscular dystrophy, cholestasis, aldosteronism, hypertension, airway mucin secretion)	21 records from the initial pool
Channel named in title but not measured	Records retrieved by acronym collision
Expression correlation with no functional or genetic evidence, where a stronger source existed for the same claim	Applied case by case
Review used as sole support for a precise mechanistic claim when the primary paper was locatable	Applied case by case; one instance was retained and graded D at submission, and is graded C in the final version after a primary source was added (Section 4.3)

S1.4. Databases not searched

Embase, Scopus, Web of Science, ClinicalTrials.gov, WHO ICTRP, and the EPO, USPTO and WIPO patent databases were not searched, in the original submission or in revision. The controlled-vocabulary searches added in revision (S1.5) were run in PubMed and are therefore a test of the search strategy, not of the database. Statements in the main text concerning the absence of a compound class, an activator or a registered trial are statements about the literature indexed in PubMed and reachable by these strings, not about the field.

S1.5. Record counts on re-execution, and the controlled-vocabulary searches added in revision

Re-executed 22 September 2026. Free-text searches S1-S15 returned, in order: 5, 11, 10, 11, 12, 8, 64, 13, 3, 1, 1, 3307, 1, 0 and 1 records (3,448 in total). S12 is a broad surveillance sweep; it was screened by title in relevance order to the point at which no conceptually new material appeared, and was not screened exhaustively. S14 returned no records on re-execution.

Four controlled-vocabulary (MeSH) searches were added in revision, constructed independently of the free-text strings in order to test the strategy for concept coverage:

M1 "Chloride Channels"[MeSH] AND "Drug Resistance, Neoplasm"[MeSH] 45 records

M2 "Anoctamin-1"[MeSH] AND ("Drug Resistance, Neoplasm"[MeSH] OR "Radiation Tolerance"[MeSH] OR "Antineoplastic Agents"[MeSH]) 45 records

M3 "Cystic Fibrosis Transmembrane Conductance Regulator"[MeSH] AND ("Drug Resistance, Neoplasm"[MeSH] OR "Antineoplastic Agents"[MeSH]) 40 records

M4 "Chloride Channels"[MeSH] AND "Neoplastic Stem Cells"[MeSH] 21 records

Nine records met the inclusion criteria of S1.2, had not been retrieved by the free-text strategy, and were added to the manuscript. They are, by their number in the final reference list: 52 (CLIC1 and cisplatin resistance in gastric cancer), 53 (OSMR-tmCLIC1 crosstalk in glioblastoma), 77 (TMEM16A and p90RSK in head and neck cancer), 78 (ANO1 blockade in radiation-induced intestinal injury), 90 (ivacaftor with osimertinib), 91 (CFTR activation and the immune microenvironment in hepatocellular carcinoma), 122 (hemin), 123 (sophoricoside) and 124 (CLNS1A).

The retrieval of nine eligible records by a differently constructed strategy indicated that the original free-text search had under-retrieved. A further audit was therefore run before resubmission and is reported in S1.6.

S1.6. Coverage audit before resubmission

Executed 28 September 2026. The purpose was concept coverage rather than currency. Three families of search were constructed: (i) protein names absent from the

original string set, principally the tweety homologues; (ii) MeSH pairings of chloride channels with neoplastic drug resistance and with related concepts; and (iii) one protein-specific free-text search for each of the four proteins carrying the directional argument, built independently of strings S1-S15.

A1 (TTYH1[tiab] OR TTYH2[tiab] OR TTYH3[tiab] OR "tweety homolog"[tiab]) AND (cancer OR tumour OR tumor OR glioma OR carcinoma OR neoplas*)[tiab] 49 records

A2 (TTYH1[tiab] OR TTYH2[tiab] OR TTYH3[tiab]) AND (chloride[tiab] OR "anion channel"[tiab] OR cryo-EM[tiab] OR structure[tiab]) 24 records

A3 (BEST1[tiab] OR bestrophin*[tiab]) AND (cancer OR tumour OR tumor OR carcinoma OR neoplas* OR glioma)[tiab] 24 records

A4 (CLCN3[tiab] OR "ClC-3"[tiab]) AND ("drug resistance"[tiab] OR chemoresistance[tiab] OR multidrug[tiab] OR cisplatin[tiab] OR paclitaxel[tiab] OR doxorubicin[tiab]) 19 records

A5 (LRRC8*[tiab] OR SWELL1[tiab] OR VRAC[tiab] OR "volume-sensitive"[tiab] OR VSOR[tiab]) AND (cisplatin[tiab] OR platinum[tiab] OR "drug resistance"[tiab] OR chemoresistance[tiab]) AND (cancer OR tumour OR tumor OR carcinoma OR neoplas*)[tiab] 54 records

A6 (CLIC1[tiab] OR "chloride intracellular channel 1"[tiab]) AND (resistance[tiab] OR chemoresistance[tiab] OR cisplatin[tiab] OR temozolomide[tiab] OR sensitiv*[tiab]) 51 records

A7 (ANO1[tiab] OR TMEM16A[tiab] OR DOG1[tiab]) AND ("drug resistance"[tiab] OR chemoresistance[tiab] OR cisplatin[tiab] OR radioresist*[tiab] OR radiotherapy[tiab] OR trastuzumab[tiab] OR osimertinib[tiab]) 58 records

A8 CFTR[tiab] AND (cancer OR tumour OR tumor OR carcinoma OR glioma)[tiab] AND (resistance[tiab] OR chemoresistance[tiab] OR cisplatin[tiab] OR "5-fluorouracil"[tiab] OR sensitiv*[tiab]) 114 records

Fourteen records met the inclusion criteria of S1.2 and had not been retrieved by either earlier strategy. They are, by their number in the final reference list: 4, 5, 6 and 7 (four prior reviews of chloride transport and treatment response, cited in the Introduction to position this review against them); 35 (Weylandt et al., the primary source for ClC-3-dependent organellar sequestration, which changed the grade of that claim in Table 2 from D to C); 42 (Siemer et al., VRAC and cisplatin resistance in head and neck cancer) and 43 (Feldman et al., NPEPPS-VRAC and platinum sensitivity), both supporting the direction argued in Section 5.1; 44, 45 and 46 (Sørensen et al., Zhang et al. and Otsuka et al.), which report LRRC8A behaving in the opposite direction for non-platinum agents and are set out as contrary evidence in Section 5.1; 58 (Han et al.), which supplies independent replication of the ClC-3 autophagy claim; 92 (Zhu et al.), CFTR-dependent cisplatin resistance in prostate cancer, a second tissue in which the CFTR direction reverses; and 126 and 127 (Sukalskaia et al. and Li et al.), the cryo-electron microscopy studies that contest the channel assignment of the tweety homologues.

One further record, 125 (Bae et al., TTYH1 and TTYH2 as LRRC8A-independent volume-regulated anion channels), was added at the same stage as a scope boundary and is discussed in Section 9. One record cited in the submitted manuscript, a bioRxiv preprint on albumin binding by VRAC blockers, was removed from the synthesis because it had not completed peer review; it is accordingly absent from Table S2.

Two rounds of independently constructed searching both returned eligible records. The original free-text strategy therefore under-retrieved substantially rather than

marginally, and the reference set should be read as sufficient for the directional argument rather than as exhaustive of the field. This is stated in Section 9 of the main text.

Table S2. Reference verification log

All 127 cited works, numbered as in the final reference list. DOI-to-PMID resolution succeeded for every record. Every record was screened for retractions and expressions of concern through the PubMed publication-type and comments-corrections fields; none is retracted and none carries an expression of concern. Three carry corrections: record 49 (affiliation of one author, J Exp Clin Cancer Res 2026, 45, 18), record 84 (addition of an omitted co-author, Oncogene 2017, 36, 3504) and record 35 (Mol Cancer Ther 2008, 7, 2261). The first two were obtained and read and neither affects the claim for which the record is cited. Verification level is given per record: "Full text" where the full text was examined and "Abstract" where verification rests on the official abstract. The fifteen records added after the controlled-vocabulary round — those numbered 4, 5, 6, 7, 35, 42, 43, 44, 45, 46, 58, 92, 125, 126 and 127 — were verified at abstract level.

#	PMID	Year	Journal	Type	Verification	Self-cit.
1	36497413	2022	Cancers (Basel)	Review	Abstract	
2	30893776	2019	Cancers (Basel)	Review	Abstract	
3	41596760	2026	Int J Mol Sci	Review	Abstract	
4	26569161	2015	Channels (Austin)	Review	Abstract	
5	31196606	2019	Curr Top Membr	Review	Abstract	
6	31129471	2019	Cell Calcium	Review	Abstract	
7	35054564	2021	Membranes (Basel)	Review	Abstract	
8	42450047	2026	Int J Mol Sci	Review	Full text	Yes
9	18772398	2008	Science	Primary	Abstract	
10	20006941	2009	Biophys J	Review	Abstract	
11	23532839	2013	J Biol Chem	Primary	Abstract	
12	25383531	2014	Nature	Primary	Abstract	
13	27295354	2016	Curr Opin Struct Biol	Review	Abstract	
14	21642943	2011	Acta Pharmacol Sin	Review	Abstract	
15	24692353	2014	Physiol Rev	Review	Abstract	
16	25451786	2015	J Mol Biol	Review	Abstract	
17	29236691	2017	Nature	Primary	Abstract	
18	33741412	2021	J Mol Biol	Review	Abstract	
19	35792944	2024	Handb Exp Pharmacol	Review	Abstract	
20	38657371	2024	Cell Calcium	Review	Abstract	
21	31061173	2019	EMBO J	Primary	Abstract	
22	23021219	2012	Cell	Primary	Abstract	
23	26417084	2015	Proc Natl Acad Sci U S A	Primary	Abstract	
24	27634832	2016	Arterioscler Thromb Vasc Biol	Primary	Abstract	
25	24790029	2014	Science	Primary	Abstract	
26	24725410	2014	Cell	Primary	Abstract	
27	31091194	2019	Biol Chem	Review	Abstract	
28	38007271	2023	Curr Top Membr	Review	Abstract	
29	25421907	2015	Oncol Rep	Review	Abstract	
30	31636470	2019	World J Gastroenterol	Review	Abstract	
31	27682822	2017	J Cell Physiol	Review	Abstract	
32	32257055	2020	Comput Struct Biotechnol J	Review	Abstract	
33	26530471	2015	EMBO J	Primary	Abstract	
34	28121479	2017	Channels (Austin)	Primary	Abstract	
35	17363491	2007	Mol Cancer Ther	Primary	Abstract	

#	PMID	Year	Journal	Type	Verification	Self-cit.
36	30230544	2019	J Cell Physiol	Primary	Abstract	
37	36069232	2022	Oncol Rep	Primary	Abstract	
38	24115360	2013	J Natl Cancer Inst	Primary	Abstract	
39	26429879	2015	Oncotarget	Primary	Abstract	
40	36467895	2022	Cancer Res Commun	Primary	Full text	
41	41053424	2025	Commun Biol	Primary	Abstract	
42	34638315	2021	Cancers (Basel)	Primary	Abstract	
43	39671496	2024	Sci Adv	Primary	Abstract	
44	27112899	2016	J Inorg Biochem	Primary	Abstract	
45	37313175	2023	Heliyon	Primary	Abstract	
46	41898509	2026	Int J Mol Sci	Primary	Abstract	
47	25361004	2014	Oncotarget	Primary	Abstract	
48	30186163	2018	Front Pharmacol	Primary	Abstract	
49	41276810	2025	J Exp Clin Cancer Res	Primary	Full text	
50	42471757	2026	Mol Ther	Primary	Abstract	
51	35135603	2022	J Exp Clin Cancer Res	Primary	Abstract	
52	37104099	2024	Anticancer Drugs	Primary	Abstract	
53	42177207	2026	Signal Transduct Target Ther	Primary	Abstract	
54	42567486	2026	Biochem Pharmacol	Primary	Abstract	
55	32587127	2020	Korean J Physiol Pharmacol	Primary	Abstract	Yes
56	40907802	2026	Biochim Biophys Acta Mol Basis Dis	Primary	Abstract	
57	37394009	2023	J Biol Chem	Primary	Full text	
58	35413227	2022	Can J Physiol Pharmacol	Primary	Abstract	
59	42630437	2026	Front Bioinform	Primary	Abstract	
60	15215166	2004	Am J Pathol	Primary	Abstract	
61	34607443	2022	Rev Esp Enferm Dig	Primary	Abstract	
62	26305547	2015	PLoS One	Primary	Full text	
63	31050728	2019	Carcinogenesis	Primary	Abstract	
64	30243029	2019	Int J Cancer	Primary	Abstract	
65	24401903	2014	Mol Med Rep	Primary	Abstract	
66	33755783	2021	J Cancer Res Clin Oncol	Primary	Abstract	
67	32380794	2020	Cells	Primary	Abstract	
68	25961581	2015	PLoS One	Primary	Abstract	
69	25823819	2015	Oncotarget	Primary	Abstract	
70	31182586	2019	Proc Natl Acad Sci U S A	Primary	Abstract	
71	36912612	2023	Cancer Res	Primary	Abstract	
72	37264829	2023	Cancer Res	Comment	Abstract	
73	34221860	2021	Acta Pharm Sin B	Review	Abstract	
74	39789065	2025	Sci Rep	Primary	Full text	
75	40356890	2025	Front Immunol	Review	Abstract	
76	40300686	2025	Int J Biol Macromol	Review	Abstract	
77	38373988	2024	BMC Cancer	Primary	Abstract	
78	40210148	2026	J Adv Res	Primary	Abstract	
79	35589730	2022	Nat Commun	Primary	Abstract	
80	39739807	2025	Proc Natl Acad Sci U S A	Primary	Full text	
81	32326161	2020	Int J Mol Sci	Review	Abstract	
82	24998896	2014	Scand J Gastroenterol	Primary	Abstract	
83	42453160	2026	JGH Open	Primary	Abstract	
84	26751771	2016	Oncogene	Primary	Abstract	
85	35743652	2022	J Pers Med	Review	Abstract	
86	42605004	2026	Cell Prolif	Review	Abstract	

#	PMID	Year	Journal	Type	Verification	Self-cit.
87	42162313	2026	Acta Pharmacol Sin	Primary	Abstract	
88	35715537	2022	J Cancer Res Clin Oncol	Primary	Abstract	
89	39437760	2024	Am J Physiol Lung Cell Mol Physiol	Primary	Abstract	
90	39627385	2025	Acta Pharmacol Sin	Primary	Abstract	
91	42270598	2026	Cell Death Dis	Primary	Abstract	
92	28592122	2017	Neoplasma	Primary	Abstract	
93	32463592	2020	J Cell Mol Med	Primary	Abstract	
94	20103641	2010	Cancer Res	Primary	Abstract	
95	41080742	2025	Biochem Biophys Rep	Review	Full text	
96	42092944	2026	Cell Commun Signal	Review	Abstract	
97	38756246	2024	Open Med (Wars)	Primary	Abstract	
98	33476655	2021	Eur J Pharmacol	Primary	Abstract	
99	25808443	2015	Biomark Med	Primary	Abstract	
100	30191737	2018	Biomark Med	Primary	Abstract	
101	36872133	2023	Eur Urol	Meta-analysis	Abstract	
102	19495750	2010	Cancer Immunol Immunother	Primary	Abstract	
103	33940566	2021	Tissue Cell	Review	Abstract	
104	39923095	2025	Cell Commun Signal	Primary	Abstract	
105	39978863	2025	J Pathol	Primary	Abstract	
106	26317553	2015	Oncotarget	Primary	Abstract	
107	32227267	2020	Ann Surg Oncol	Primary	Abstract	
108	32206949	2020	Ann Surg Oncol	Primary	Abstract	
109	29024940	2017	Br J Cancer	Primary	Abstract	
110	37979051	2024	Pflugers Arch	Primary	Abstract	
111	27219012	2016	PLoS One	Primary	Full text	Yes
112	31814333	2019	Physiol Rep	Primary	Abstract	
113	23012257	2013	Mol Pharmacol	Primary	Abstract	
114	26837888	2016	Pflugers Arch	Primary	Abstract	
115	23072356	2013	Br J Pharmacol	Primary	Abstract	
116	35102550	2022	Br J Pharmacol	Primary	Abstract	
117	42107400	2026	Biochem Biophys Res Commun	Primary	Abstract	
118	32319819	2020	Assay Drug Dev Technol	Primary	Abstract	
119	33827113	2021	Nature	Primary	Abstract	
120	37573365	2023	Nat Commun	Primary	Abstract	
121	32201246	2020	Pharmacol Res	Primary	Abstract	
122	38892219	2024	Int J Mol Sci	Primary	Abstract	
123	41014666	2025	Phytomedicine	Primary	Abstract	
124	40345428	2025	Cancer Lett	Primary	Abstract	
125	31181821	2019	Cells	Primary	Abstract	
126	34385445	2021	Nat Commun	Primary	Abstract	
127	34824283	2021	Nat Commun	Primary	Abstract	

Every record: DOI resolved = Yes; retraction and expression-of-concern screening = performed; result = none identified. Self-citations are flagged in the final column and number three.
